# Synthesis and Antiproliferative Activity of Marine Bromotyrosine Purpurealidin I and Its Derivatives

**DOI:** 10.3390/md16120481

**Published:** 2018-12-03

**Authors:** Chinmay Bhat, Polina Ilina, Irene Tilli, Manuela Voráčová, Tanja Bruun, Victoria Barba, Nives Hribernik, Katja-Emilia Lillsunde, Eero Mäki-Lohiluoma, Tobias Rüffer, Heinrich Lang, Jari Yli-Kauhaluoma, Paula Kiuru, Päivi Tammela

**Affiliations:** 1 Drug Research Program, Division of Pharmaceutical Chemistry and Technology, Faculty of Pharmacy, Viikinkaari 5 E (P.O. Box 56), University of Helsinki, FI-00014 Helsinki, Finland; chinmay.bht@gmail.com (C.B.); irene.tilli@helsinki.fi (I.T.); manuelavorac@gmail.com (M.V.); tanja.bruun@helsinki.fi (T.B.); victoria.barba@ens-lyon.fr (V.B.); nives.hribernik@gmail.com (N.H.); Eero.Maki-Lohiluoma@orion.fi (E.M.-L.); jari.yli-kauhaluoma@helsinki.fi (J.Y.-K.); paula.kiuru@helsinki.fi (P.K.); 2 Drug Research Program, Division of Pharmaceutical Biosciences, Faculty of Pharmacy, Viikinkaari 5 E (P.O. Box 56), University of Helsinki, FI-00014 Helsinki, Finland; polina.ilina@helsinki.fi (P.I.); katja-emilia.lillsunde@helsinki.fi (K.-E.L.); 3 Institute of Chemistry, Technische Universität Chemnitz, 09107 Chemnitz, Germany; tobias.rueffer@chemie.tu-chemnitz.de (T.R.); heinrich.lang@chemie.tu-chemnitz.de (H.L.)

**Keywords:** Purpurealidin I, bromotyrosines, *Pseudoceratina purpurea*, synthesis, cytotoxicity, selectivity to cancer cells

## Abstract

The first total synthesis of the marine bromotyrosine purpurealidin I (**1**) using trifluoroacetoxy protection group and its dimethylated analog (**29**) is reported along with 16 simplified bromotyrosine derivatives lacking the tyramine moiety. Their cytotoxicity was evaluated against the human malignant melanoma cell line (A-375) and normal skin fibroblast cells (Hs27) together with 33 purpurealidin-inspired simplified amides, and the structure–activity relationships were investigated. The synthesized simplified analogs without the tyramine part retained the cytotoxic activity. Purpurealidin I (**1**) showed no selectivity but its simplified pyridin-2-yl derivative (**36**) had the best improvement in selectivity (Selectivity index 4.1). This shows that the marine bromotyrosines are promising scaffolds for developing cytotoxic agents and the full understanding of the elements of their SAR and improving the selectivity requires further optimization of simplified bromotyrosine derivatives.

## 1. Introduction

Globally, cancer is the second leading cause of death and in 2018 it is estimated to lead to 9.6 million deaths [1] and malignant melanoma is one of the most life-threatening cancers due to resistance to most therapies [2]. While prevention is important, there is a continuous need for novel treatments. The marine environment provides a potential source for discovering new drug lead molecules, especially against cancer. So far, four medicinal products originating from marine ecosystems have been registered for the treatment of different kinds of cancer such as leukemia, metastatic breast cancer, and ovarian cancer [3,4].

Bromotyrosines are a large and structurally diverse group of bromine-containing marine alkaloids which have shown a variety of biological functions including antimicrobial, antiviral, antifungal and in particular, anticancer activity [5,6,7,8] Bromotyrosines are mainly isolated from the marine sponges of the order Verongida. For representative articles about secondary metabolites of Verongida order, see, for example, Fattorusso group’s work [9,10].

**Figure 1:**
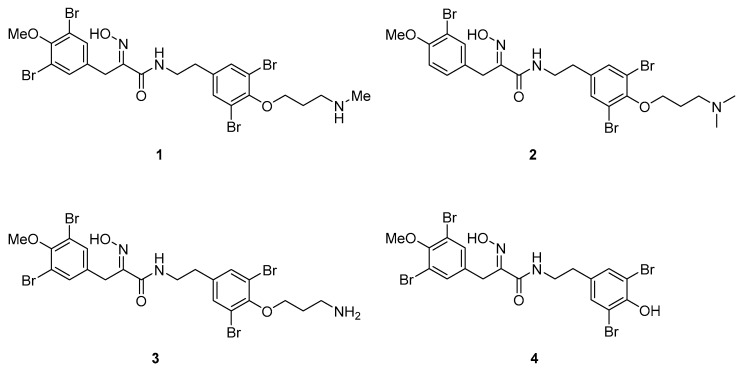
Bromotyrosines purpurealidin I (**1**), aplysamine 2 (**2**), aplysamine 4 (**3**) and JBIR-44 (**4**).

Purpurealidin I (**1**; Figure 1) together with several other bromotyrosines have been isolated from the Indian sea sponge *Pseudoceratina* (*Psammaplysilla*) *purpurea* [11]. Structurally similar aplysamine 2 (**2**) was isolated from the Australian marine sponge, *Aplysina* sp. [12]. Purpurealidin I (**1**) has been found to be cytotoxic when tested against various cancer cell lines (ovarian cancer A2780S and cisplatin-resistant variant A2780CP (SCP5), non-small cell lung cancer A549, human breast cancer MCF7 and glioma U251MG cells), as well as non-cancer cell line NIH3T3 (normal mouse fibroblasts) [6]. Two other bromotyrosines aplysamine 4 (**3**) **[13]** and JBIR-44 (**4**) **[14]** were isolated from *P. purpurea* and have been tested against human cervical carcinoma HeLa cells [5]. A comparably strong cytotoxic effect was observed and there was no difference between the compounds with a longer or shorter alkyl chain attached to the tyramine part. This presents the opportunity for the design of simplified analogs of marine bromotyrosines as the long alkyl chain does not seem to be essential for cytotoxicity. In our previous studies, simplified amide-linked bromotyrosines inspired by purpurealidin I (**1**) displayed good K_v_10.1 channel inhibition [15]. K_v_10.1 potassium channel regulates many fundamental functions in a cell, for example cell cycle progression and cellular proliferation [16]. We report here the total synthesis of the marine natural product purpurealidin I (**1**) and a related tetrabrominated analog of aplysamine 2 (**2**; also, a dimethyl analog of **1**). Medicinal chemistry strategies to simplify their structures are also outlined. Furthermore, we have evaluated these compounds for selective cytotoxic effects to skin cancer cells and discussed their structure-activity relationships.

## 2. Results

### 2.1. Chemistry

**Scheme 1:**
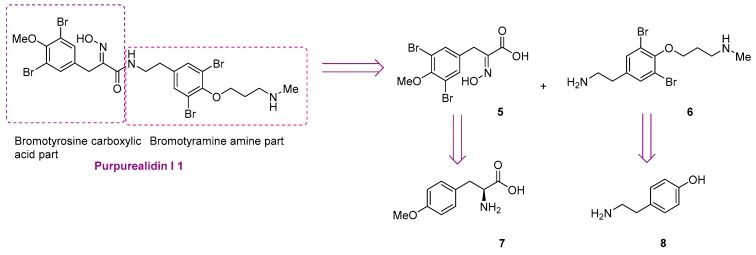
Retrosynthetic route to purpurealidin I (**1**).

The purpurealidin I (**1**) skeleton can be viewed as a secondary amide. The retrosynthetic pathway (Scheme 1) illustrated that the synthesis of the bromotyrosine carboxylic acid part could be initiated from *O*-methyltyrosine (**7**) and the corresponding amine part from tyramine (**8**).

**Scheme 2:**
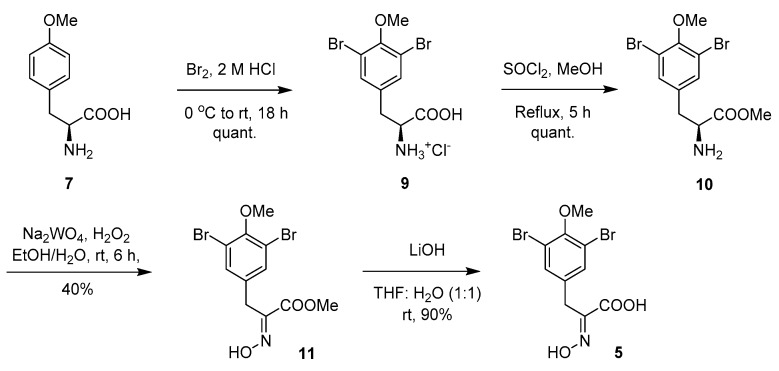
Synthesis of the bromotyrosine carboxylic acid part (**5**) [17] for the first amide coupling attempts.

For the synthesis of the bromotyrosine carboxylic acid moiety, commercially available *O*-methyltyrosine (**7**) was brominated [17] and subjected to an esterification reaction with SOCl_2_ in MeOH. The ester (**10**) was converted to oxime (**11**) using sodium tungstate and hydrogen peroxide, following a literature procedure (Scheme 2) [17,18,19,20]. The corresponding carboxylic acid subunit (**5**) was then synthesized via the LiOH-mediated hydrolysis of methyl ester (**11**) in 90% yield (See the Supplementary Information for the experimental details).

**Scheme 3:**
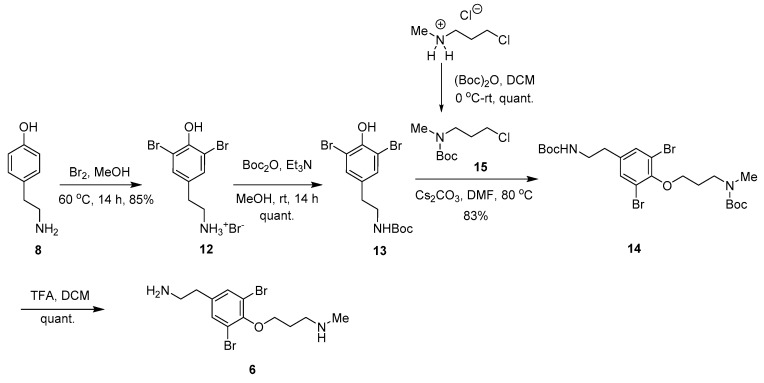
Synthesis of the tyramine derivative (**6**) for use in the first amide coupling approach.

The first attempts of amide coupling were made using the free primary amine (**6**) (Scheme 3). Tyramine (**8**) underwent bromination followed by *N*-Boc protection to give (**13**) [19,20,21,22,23] which was then subjected to *O*-alkylation with Boc-protected 3-chloro-*N*-methylpropan-1-amine (**15**) [24] to give (**14**) in 83% yield. The trifluoroacetic acid (TFA)-mediated Boc deprotection of (**14**) gave the diamine (**6**) in quantitative yield.

**Scheme 4:**
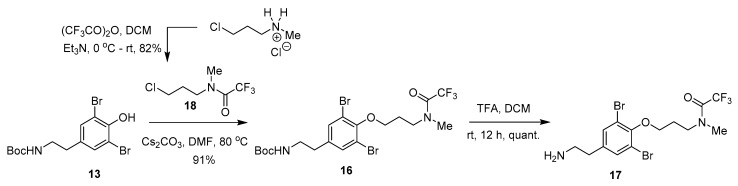
Synthesis of trifluoroacetyl protected tyramine part (**17**) [15] for the purpurealidin I (**1**) synthesis.

The direct coupling of compounds (**5**) and (**6**) with 1-ethyl-3-(3-dimethylaminopropyl)carbodiimide (EDC) was unsuccessful. This was likely due to the interfering free hydroxy group within (**5**). A condensation reaction of ester (**11**) with compound (**6**) also proved unsuccessful. Several alternative coupling conditions were tried (Supplementary Information, Table S1) Unfortunately, each resulted in the formation of inseparable mixtures with poor yields of the desired product, purpurealidin I (**1**), as determined by ^1^H NMR and LC-MS analyses. After several additional approaches proved unsuccessful (data not shown), trifluoroacetyl was found to be a suitable protecting group for the secondary amine (Scheme 4). The Boc-protected bromo tyramine (**13**) was *O*-alkylated with (**18**) to produce (**16**). Treatment of (**16**) with TFA led to the selective Boc deprotection and led to the formation of the desired target amine (**17**) in 77% overall yield [15].

**Scheme 5:**
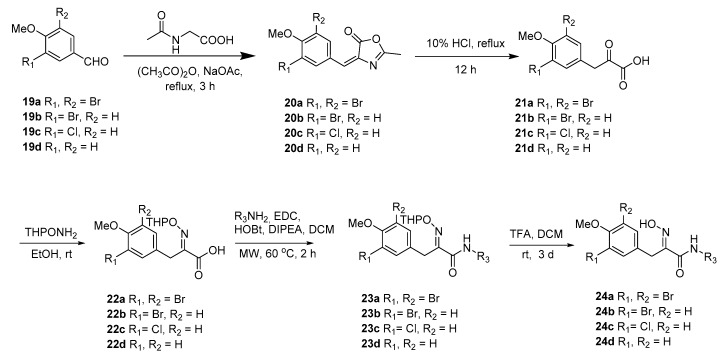
Synthesis of the purpurealidin I (**1**) carboxylic part (**22a**) and a route to the simplified hydroxyimino propanamides (**24a**–**d**), R_3_ substituents are given in Table 1.

An initial attempt to synthesize the immediate precursor of purpurealidin I (**1**) by direct condensation of (**17**) with hydroxylamine ester (**11**) was unsuccessful. This was likely due to the presence of the hydroxylamino moiety. We then decided to introduce the free hydroxylamino group after the amide coupling. The bromotyrosine carboxylic acid fragment (**22a**) was prepared via the Erlenmeyer–Plöchl azlactone method (Scheme 5) in 84% overall yield [25,26]. This procedure was also used for the preparation of the carboxylic acid fragments (**22b**–**d**) in the mono-brominated, mono-chlorinated and non-halogenated simplified derivatives (**41**–**45**). Reddy et al. have reported the synthesis of methyl carbamate containing bromotyrosine purpuramine K leaving the tetrahydropyranyl (THP) group to the molecule [22].

**Scheme 6:**
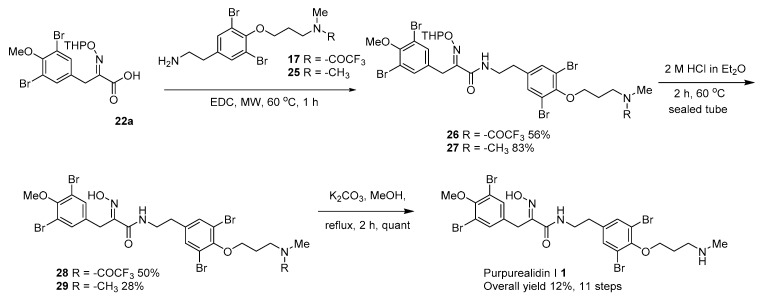
Synthesis of purpurealidin I (**1**) and aplysamine 2 analog (**29**).

The synthesis of purpurealidin I (**1**), began with the bromination of *p*-hydroxybenzaldehyde, followed by methylation to give (**19a**). The requisite azlactone (**20a**) was then prepared by the condensation of (**19a**) with *N*-acetylglycine (Scheme 5) and subjecting the resulting product to hydrolysis using a 10% aqueous solution of HCl to give pyruvic acid (**21a**)**.** Compound (**21a**) was then converted into the THP-protected oxime (**22a**) by reaction with *O*-(tetrahydropyran-2-yl)-hydroxylamine (THPONH_2_). The crude oxime was then subjected to EDC coupling with (**17**) under microwave conditions to produce (**26**) in a moderate yield (56%; Scheme 6). The THP group was removed with a 2 M solution of HCl in Et_2_O to give the free oxime (**28**). Trifluoroacetyl mediated deprotection of (**28**) using MeOH/K_2_CO_3_ resulted in purpurealidin I (**1**) in an overall yield of 12% (11 steps). The aplysamine 2 tetrabromo derivative (**29**)**,** with a dimethylamino moiety at the tyramine fragment, was synthesized using purpurealidin E (**25**) [15,27] in the coupling with (**22a**) (Scheme 6).

The simplified derivatives of purpurealidin I (**1**) were prepared in an analogous manner (Scheme 5) with the appropriate anilines and benzylamines (Table 1) followed by THP deprotection. The yields of the amide couplings ranged from 19–87%. The THP deprotection was achieved using TFA for the various simplified derivatives of (**1**), as heating with 2 M HCl in Et_2_O proved sluggish. Several different conditions were attempted in the synthesis of compound (**31**) (see Experimental Section 4.1.2). After purification on silica gel, the yields of the final hydroxyimino propanamides (**30**–**45**) ranged from 13–50%.

Before finalizing the purpurealidin I (**1**) synthesis, several synthetically simpler amide analogs containing the tyramine fragment and a series of compounds with substituted phenyl rings (Ar in Table 2) instead of the bromotyrosine part were synthesized and screened. The synthesis of these simplified amide derivatives (**46**–**78**) (Table 2) have previously been reported by our group [15].

### 2.2. Stereochemistry

**Figure 2:**
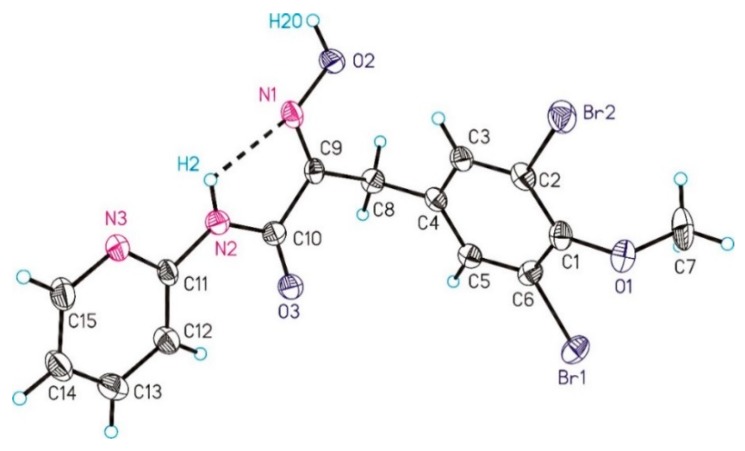
ORTEP representation (50% probability ellipsoids) of the molecular structure of (**36**). The CHCl_3_ molecule as a packing solvent has been omitted for clarity.

It has previously been reported that the configuration of the *N*-oxime can be determined by analysis of the ^13^C NMR C-8 carbon shifts, which are known to be approximately 26–30 ppm (*E* configuration) or over 35 ppm (*Z* configuration) [28]. The reported X-ray structure of a disulfide-bridged psammaplin A analog supported this observation [29]. We, therefore, expect the stereochemistry of all bromotyrosines synthesized herein to be *E*, since the benzylic C-8 shifts in ^13^C NMR were around 28 ppm. This was supported by the crystal structure of pyridin-2-yl analog (**36**), which was determined by the single crystal X-ray diffraction (Figure 2; see Supplementary Information for experimental details and crystallographic data). An intramolecular hydrogen bond between the secondary amide hydrogen atom H2 and the lone electron pair of oxime nitrogen atom N1 could explain the observed *E* geometry of the oxime.

### 2.3. Biological Activity

The cytotoxicity of the synthetic purpurealidin I (**1**) and compounds (**29**–**78**) against cancer cells was primarily evaluated in human malignant melanoma A-375 cell line at the single concentration of 50 µM (Table 3). The compounds demonstrating over 80% cytotoxicity were selected for confirmatory dose-response experiments in the same cell line, and CC_50_ (cytotoxic concentration that caused death of 50% cells) was calculated (Table 3). We furthermore aimed to evaluate the potential of the compounds to selectively perturb the growth of cancer cells. Therefore, the compounds with the highest cytotoxic activities (CC_50_ below 15 µM) were studied for cytotoxicity in normal human fibroblast cell line Hs27 (Table 3). The degree of selectivity towards cancer cells can be expressed by selectivity index (SI). High values show selectivity towards cancer cells, while values <2 suggest general cytotoxicity of the compound [30]. Camptothecin, a naturally occurring alkaloid with known high selectivity to cancer cells (SI 92.3, Table 3) was used as a positive control. Most of the compounds demonstrated general cytotoxicity (SI < 2, Table 3). The highest selectivity to cancer cells (SI 4.1, Table 3) was shown for the compound (**36**).

## 3. Discussion

### SAR

The observed melanoma cell line activity for the compounds was in the range of 4–43 µM (CC_50_ in A-375 cells, Table 3). Both Purpurealidin I (**1**) and its dimethylated analog (**29**) showed cytotoxicity to melanoma cells (CC_50_ 4.3 and 6.3 μM, respectively). Maintaining the hydroxylamine linker but replacing the longer tyramine fragment with the aniline moiety (e.g., (**36**) 4.7 μM) or benzyl amine with a one-carbon chain (e.g., (**31**) 12.4 μM) retained the activity. This indicates that the tyramine part is not essential for the activity. The cytotoxic activity of the pyridine derivatives with a hydroxyimino amide was found to be in the order of pyridin-2-yl (**36**) > pyridin-3-yl (**37**) > pyridin-4-yl (***3*8**). Furthermore, pyridin-3-yl methyl amide (**39**) retained the activity. Two bromine atoms in the tyrosine part seem to be essential for the cytotoxicity since the non-halogenated (**44**) or mono-halogenated (**42** and **45**) pyridin-2-yls were inactive. However, compound (**41**) with two mono-brominated *p*-methoxyphenyl rings showed activity even though the mono-brominated (**42**), with pyridin-2-yl group, was inactive. This may imply a different binding mode exists for this bis mono-brominated *p*-methoxyphenyl compound. However, both structural data of the binding sites and the mechanism of action are currently unknown, and cytotoxicity of these compounds cannot be accounted for.

The synthesis of the simplified amides with the tyramine end allowed for the more feasibly exploration of the aromatic substituents at the tyrosine part of the molecule. The CC_50_ (A-375 cells) values were retained with the best compounds, monomethylamino *m*-dichloro-*p*-methoxy (**65**) (6.4 μM) and *m*-iodo-*p*-methoxy (**74**) (6.2 μM). Non-halogenated amides in the tyrosine part (**59**) and (**61**) were also inactive, and *o*-bromo (**46**) or *o*-fluoro (**57**) substitution resulted in low activity. The CC_50_ values did not show a significant difference when compared the monomethylated amines at the end of the tyramine part to the dimethylated ones (e.g., CC_50_ in A-375 cells 6.2 μM for (**74**) and 8.4 μM for (**73**)). Replacement of the amino group in the tyramine end to isopropyl in compounds (**49**) and (**52**), as well as the addition of the morpholine moiety in (**55**) resulted in the loss of the activity.

Purpurealidin I (**1**) and its dimethylated analog (**29**) showed no selectivity in cytotoxicity between melanoma A-375 cell line and normal human fibroblast cell line Hs27 (SI 1.2 and 0.7, respectively, Table 3). Changing the linker from hydroxyimino amide to amide did not improve the selectivity, and different aromatic substitution on the tyrosine fragment also had no effect (SI’s varied between 0.5–1.3) However, when the longer tyramine part was replaced with directly attached aniline, some improvement in the selectivity was observed (**1** SI 1.2 compared to **33** SI 2.0 or **36** SI 4.1). The pyridin-2-yl compound **36** displayed the best, albeit only moderate, selectivity (SI 4.1, Table 3). 

## 4. Materials and Methods

### 4.1. Synthesis Experimental

#### 4.1.1. General

All reactions were carried out using commercially available starting materials unless otherwise stated. The melting points were measured using a Stuart SMP40 automated melting point apparatus and are uncorrected. ^1^H NMR (300 MHz) and ^13^C NMR (75 MHz) spectra were measured in CDCl_3_, *d*_6_-DMSO, CD_3_OD, or *d*_6_-acetone at room temperature and were recorded on a Varian Mercury *Plus* 300 spectrometer or Bruker AV400 MHz NMR with smart probe. Chemical shifts (*δ*) are given in parts per million (ppm) relative to the ^1^H and ^13^C NMR reference solvent signals (CDCl_3_: 7.26 and 77.16 ppm; CD_3_OD: 3.31 and 49.00 ppm; *d*_6_-DMSO: 2.50 ppm and 39.52; *d*_6_-acetone: 2.05 and 29.84 ppm). Multiplicities are indicated by s (singlet), br s (broad singlet), d (doublet), dd (doublet of doublet), ddd (doublet of doublet of doublets), t (triplet), dt (doublet of triplets), q (quartet) and m (multiplet). The coupling constants *J* are quoted in Hertz (Hz). LC-MS and HRMS-spectra were recorded using a Waters Acquity UPLC^®^-system (Milford MA, USA) with Acquity UPLC^®^ BEH C18 column (1.7 µm, 50 × 2.1 mm, Waters, Wexford, Ireland) with Waters Synapt G2 HDMS (Milford MA, USA) with the ESI (+), high resolution mode. The mobile phase consisted of H_2_O (A) and acetonitrile (B) both containing 0.1% HCOOH. Microwave synthesis were performed in sealed tubes using Biotage Initiator+ instrument equipped with an external IR sensor. The flash chromatography was performed with Biotage SP1 flash chromatography purification system with 254 nm UV-detector or Biotage Isolera™ Spektra Systems with 200–800 nm UV-detector using SNAP 10, 25, 50 or 100 g cartridges (Uppsala, Sweden). The TLC-plates were provided by Merck (Darmstadt, Germany, Silica gel 60-F254) and visualization of the amine compounds was done using ninhydrin staining and THP ethers with vanillin staining.

#### 4.1.2. Experimental Procedures

##### General Procedure for the Formation of Azlactones

Aldehyde **19a**–**d**, acetylglycine (1.5 equiv.) and anhyd. NaOAc (1.5 equiv.) were dissolved in Ac_2_O (10–15 mL) and the reaction mixture was stirred at 80 °C overnight. Afterwards, the reaction mixture was cooled to room temperature and poured into water (50 mL). The formed precipitate was filtered, washed with water (4 × 20 mL) and dried in vacuo. The obtained crude product **20a**–**d** was used in the subsequent step without further purification.


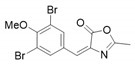


*4-(3,5-Dibromo-4-methoxybenzylidene)-2-methyloxazol-5(4H)-one* (**20a**).

3,5-Dibromo-4-methoxybenzaldehyde **19a** (1.57 g, 5.33 mmol) gave **20a** as a grey solid (1.94 g, 97%). ^1^H NMR (400 MHz, CDCl_3_) *δ* 8.26 (s, 2H), 6.92 (s, 1H), 3.93 (s, 3H), 2.43 (s, 3H). ^1^H NMR is in accordance with the literature [26].


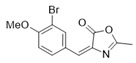


*4-(3-Bromo-4-methoxybenzylidene)-2-methyloxazol-5(4H)-one* (**20b**).

3-Bromo-4-methoxybenzaldehyde **19b** (2.00 g, 9.30 mmol) gave **20b** as a yellow solid (2.70 g, 98%) ^1^H NMR (400 MHz, CDCl_3_) *δ* 8.43 (d, *J* = 2.1 Hz, 1H), 7.98–7.94 (m, 1H), 7.02 (s, 1H), 6.95 (d, *J* = 8.6 Hz, 1H), 3.96 (s, 3H), 2.41 (d, *J* = 0.7 Hz, 3H). ^1^H NMR is in accordance with the literature [25].


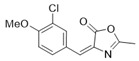


*4-(3-Chloro-4-methoxybenzylidene)-2-methyloxazol-5(4H)-one* (**20c**). 

3-Chloro-4-methoxybenzaldehyde **19c** (2.43 g, 14.3 mmol) gave **20c** as a yellow solid (2.66 g, 74%). ^1^H NMR (400 MHz, CDCl_3_) *δ* 8.28 (d, *J* = 2.1 Hz, 1H), 7.89 (dd, *J* = 8.6, 2.1 Hz, 1H), 7.02 (s, 1H), 6.98 (d, *J* = 8.6 Hz, 1H), 3.97 (s, 3H), 2.41 (s, 3H).


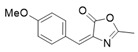


*4-(4-Methoxybenzylidene)-2-methyloxazol-5(4H)-one* (**20d**).

4-Methoxybenzaldehyde **19d** (3.03 g, 22.3 mmol) gave **20d** as a yellow solid (2.21 g, 46%). ^1^H NMR (400 MHz, CDCl_3_) *δ* 8.06 (d, *J* = 8.5 Hz, 2H), 7.11 (s, 1H), 6.96 (d, *J* = 8.9 Hz, 2H), 3.87 (s, 3H), 2.39 (s, 3H).

##### General Method for the Hydrolysis of Azlactones

Azlactone **20a**–**d** was dissolved in a 10% solution of HCl in H_2_O (30 mL). A capillary tube was introduced in the flask to allow the reflux despite a solid layer forming while heating. The reaction mixture was refluxed overnight. The reaction mixture was cooled to room temperature and poured into cold water (2 × 10 mL). The resulting precipitate was filtered, washed with water (4 × 20 mL) and dried in vacuo. The obtained crude product **21a**–**d** was used in the subsequent step without further purification.


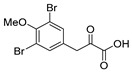


*3-(3,5-Dibromo-4-methoxyphenyl)-2-oxopropanoic acid* (**21a**)

Azlactone **20a** (1.74 g, 4.63 mmol) gave acid **21a** as a yellowish solid (1.46 g, 89%). ^1^H NMR (400 MHz, *d*_6_-DMSO) *δ* 8.06 (s, 2H), 3.80 (s, 3H), 3.32 (s, 2H); ^13^C NMR (101 MHz, *d*_6_-DMSO) *δ* 165.7, 151.8, 143.3, 134.5, 132.9, 117.3, 106.1, 60.5. NMR showed the enol tautomer. ^1^H NMR is in accordance with the literature [25].


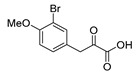


*3-(3-Bromo-4-methoxyphenyl)-2-oxopropanoic acid* (**21b**)

Azlactone **20b** (3.33 g, 9.11 mmol) gave **21b** as a dark red solid (1.89 g, 79%). ^1^H NMR (400 MHz, *d*_6_-DMSO) *δ* 9.26 (bs, 1H), 8.10 (d, *J* = 2.1 Hz, 1H), 7.69–7.64 (m, 1H), 7.10 (d, *J* = 8.7 Hz, 1H), 6.35 (s, 1H), 3.85 (s, 3H). NMR showed the enol tautomer. ^1^H NMR is in accordance with the literature [25].


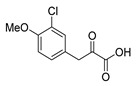


*3-(3-Chloro-4-methoxyphenyl)-2-oxopropanoic acid* (**21c**)

Azlactone **20c** (2.66 g, 10.6 mmol) gave **21c** as a brown solid (1.92 g, 79%). ^1^H NMR (400 MHz, CD_3_OD) *δ* 7.91 (d, *J* = 2.1 Hz, 1H), 7.59 (ddd, *J* = 8.6, 2.1, 0.5 Hz, 1H), 7.03 (d, *J* = 8.7 Hz, 1H), 6.40 (s, 1H), 3.89 (s, 3H). NMR showed the enol tautomer.


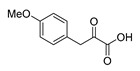


*3-(4-Methoxyphenyl)-2-oxopropanoic acid* (**21d**)

Azlactone **20d** (2.21 g, 10.2 mmol) gave product **21d** as a brown solid (1.78 g, 90%). ^1^H NMR (400 MHz, CDCl_3_) *δ* 7.75 (d, *J* = 8.7 Hz, 2H), 6.92 (d, *J* = 8.9 Hz, 2H), 6.64 (s, 1H), 3.84 (s, 3H). NMR showed the enol tautomer. ^1^H NMR is in accordance with the literature [31].

##### General Procedure for THP-Protection

Carboxylic acid **21a**–**d** and THPONH_2_ (2 equiv.) were dissolved in dry ethanol (15–20 mL). The reaction mixture was stirred at room temperature for 18–48 h under argon atmosphere. The reaction mixture was concentrated under reduced pressure and then EtOAc (20 mL) was added to the residue. The organic layer was washed with a 2 M solution of HCl in H_2_O (2 × 20 mL). The aqueous layer was extracted back with EtOAc (2 × 10 mL). The combined organic layers were dried over Na_2_SO_4_, filtered and concentrated in vacuo. The obtained crude product **22a**–**d** was used in the subsequent step without further purification.


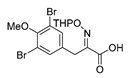


*(E)-3-(3,5-Dibromo-4-methoxyphenyl)-2-[[(tetrahydro-2H-pyran-2-yl)oxy]imino]propanoic acid* (**22a**)

Carboxylic acid **21a** (1.16 g, 3.30 mmol) and THPONH_2_ (0.97 g, 8.3 mmol, 2 equiv.) were used. The product was purified by column chromatography, Biotage SNAP Cartridge KP-Sil 25 g, gradient elution: (DCM/MeOH, 0→10%) to give **22a** as a yellow oil (1.44 g, 97%). ^1^H NMR (400 MHz, CDCl_3_) *δ* 7.48 (s, 2H), 5.50–5.41 (m, 1H), 3.95–3.88 (m, 2H), 3.84 (s, 3H), 3.69–3.54 (m, 2H), 1.93–1.83 (m, 2H), 1.77–1.65 (m, 2H), 1.64–1.58 (m, 2H). ^13^C NMR (101 MHz, CDCl_3_) *δ* 163.7, 163.3, 153.2, 149.9, 133.6, 118.2, 102.2, 62.4, 60.8, 29.6, 28.1, 24.9, 18.6.


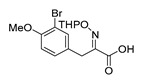


*(E)-3-(3-Bromo-4-methoxyphenyl)-2-[[(tetrahydro-2H-pyran-2-yl)oxy]imino]propanoic acid* (**22b**)

Carboxylic acid **21b** (2.00 g, 7.32 mmol) and THPONH_2_ (1.71 g, 14.6 mmol, 2 equiv.) were used. **22b** was obtained as an oil (2.74 g, quant.). ^1^H NMR (400 MHz, CDCl_3_) *δ* 7.54 (d, *J* = 2.2 Hz, 1H), 7.23 (dd, *J* = 2.2, 8.5 Hz, 1H), 6.81 (d, *J* = 8.5 Hz, 1H), 5.46 (d, *J* = 3.1 Hz, 1H), 3.94–3.87 (m, 2H), 3.86 (s, 3H), 3.69–3.65 (m, 2H), 1.91–1.81 (m, 2H), 1.76–1.66 (m, 2H), 1.65–1.56 (m, 2H).


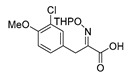


*(E)-3-(3-Chloro-4-methoxyphenyl)-2-[[(tetrahydro-2H-pyran-2-yl)oxy]imino]propanoic acid* (**22c**)

Carboxylic acid **21c** (1.86 g, 8.13 mmol) and THPONH_2_ (1.90 g, 16.3 mmol, 2 equiv.) were used. **22c** was obtained as a yellow oil (3.05 g, quant). ^1^H NMR (400 MHz, CDCl_3_) *δ* 7.36 (d, *J* = 2.2 Hz, 1H), 7.18 (dd, *J* = 8.4, 2.2 Hz, 1H), 6.84 (d, *J* = 8.4 Hz, 1H), 5.47 (d, *J* = 3.1 Hz, 1H), 3.87 (s, 3H), 3.86 (s, 2H), 3.63 (dd, *J* = 7.6, 3.4 Hz, 2H), 1.91–1.82 (m, 2H), 1.77–1.66 (m, 2H), 1.65–1.52 (m, 2H).


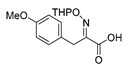


*(E)-3-(4-Methoxyphenyl)-2-[[(tetrahydro-2H-pyran-2-yl)oxy]imino]propanoic acid* (**22d**)

Carboxylic acid **21d** (1.73 g, 7.98 mmol) and THPONH_2_ (1.87 g, 16.0 mmol, 2 equiv.) were used. **22d** was obtained as a yellow oil (2.78 g, quant). ^1^H NMR (400 MHz, CDCl_3_) *δ* 7.23 (d, *J* = 8.7 Hz, 2H), 6.81 (d, *J* = 8.7 Hz, 2H), 5.47–5.43 (t, 1H), 3.87 (s, 2H), 3.76 (s, 3H), 3.67–3.59 (m, 2H), 1.93–1.80 (m, 2H), 1.72–1.65 (m, 2H), 1.60–1.51 (m, 2H).


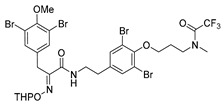


*(E)-N-[3,5-Dibromo-4-[3-(2,2,2-trifluoro-N-methylacetamido)propoxy]phenethyl]-3-(3,5-dibromo-4-methoxyphenyl)-2-[[(tetrahydro-2H-pyran-2-yl)oxy]imino]propanamide* (**26**)

A 20 mL Biotage MW tube was charged with carboxylic acid **22a** (0.24 g, 0.53 mmol), amine **17** [15] (0.25 g, 0.53 mmol), EDC·HCl (0.15 g, 0.79 mmol, 1.5 equiv.), HOBt (0.11 g, 0.79 mmol, 1.5 equiv.), DIPEA (0.10 g, 0.79 mmol, 1.5 equiv.) and dry DCM (15 mL). The reaction was MW irradiated for 2 h at 60 °C (5 bar). The reaction mixture was diluted with DCM (25 mL), washed with water (2 × 15 mL) and a 1 M solution of HCl in H_2_O (15 mL). The organic layer was dried over Na_2_SO_4_ and concentrated in vacuo. The crude product was purified by flash chromatography twice, 25 g, gradient elution: (heptane/EtOAc, 0→40%) to **23** as a pale yellow viscous liquid (0.16 g, 34%). ^1^H NMR (400 MHz, CDCl_3_) *δ* 7.50 (s, 2H), 7.34 and 7.33 (2 s, 2H, rotamers ratio 2:1), 6.92 (t, *J* = 6.2 Hz, 1H), 5.38 (t, *J* = 2.8 Hz, 1H), 4.06–4.00 (m, 2H), 3.93 (d, *J* = 13.1 Hz, 1H), 3.84 (s, 3H), 3.83 (d, *J* = 13.1 Hz, 1H), 3.80–3.72 (m, 2H), 3.65–3.54 (m, 3H), 3.43 (ddt, *J* = 12.9, 8.2, 6.6 Hz, 1H), 3.23 (m) and 3.11 (m) N-CH_3_ rotamers 2:1, 2.77 (td, *J* = 7.3, 1.7 Hz, 2H), 2.23–2.09 (m, 2H), 1.89–1.54 (m, 6H). 


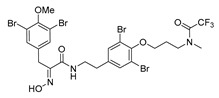


*(E)-N-[3,5-Dibromo-4-[3-(2,2,2-trifluoro-N-methylacetamido)propoxy]phenethyl]-3-(3,5-dibromo-4-methoxyphenyl)-2-(hydroxyimino)propanamide* (**28**)

THP ether **26** (0.14 g, 0.16 mmol), a 2 M solution of HCl in Et_2_O (4 mL), dry DCM (4 mL) and dry MeOH (0.3 mL) were added to a 20-mL sealed tube and heated in an oil bath at 70 °C for 3 h. The reaction mixture was then concentrated in vacuo. The crude product was purified by column chromatography, Biotage SNAP Cartridge KP-Sil 10 g, gradient elution: (heptane/EtOAc, 0→30%) to give **28** as a pale yellow viscous liquid (0.068 g, 54%). ^1^H NMR (400 MHz, CD_3_OD) *δ* 7.48 (s, 2H), 7.43 and 7.42 (2 s, 2H, rotamers ratio 2:1), 4.00 (t, *J* = 6.0 Hz, 2H), 3.82 (m, 5H), 3.78–3.70 (m, 2H), 3.44 (t, *J* = 7.1 Hz, 2H), 3.24 (q) and 3.10 (m) N-CH_3_ rotamers 2:1, 2.76 (t, *J* = 7.1 Hz, 2H), 2.25–2.08 (m, 2H).


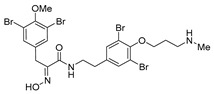


*(E)-N-[3,5-Dibromo-4-[3-(methylamino)propoxy]phenethyl]-3-(3,5-dibromo-4-methoxyphenyl)-2-(hydroxyimino)propenamide, purpurealidin I*, (**1**)

Compound **28** (0.050 g, 0.062 mmol) and K_2_CO_3_ (0.018 g, 0.12 mmol, 2.0 equiv.) in MeOH (5 mL) and H_2_O (0.5 mL) were refluxed for 2.5 h. The reaction mixture was concentrated in vacuo and partitioned between EtOAc (10 mL) and water (4 mL). The aqueous layer was back-extracted with EtOAc (10 mL). The combined organic layers ware dried over Na_2_SO_4_, filtered and the solvent was removed in vacuo to give **1**, as a pale yellow viscous liquid (0.040 -g, 91%). ^1^H NMR (300 MHz, CDCl_3_) *δ* 7.49 (s, 2H), 7.26 (s, 2H), 4.16–4.11 (m, 2H), 3.84–3.83 (m, 5H), 3.42–3.35 (m, 2H), 2.93 (t, *J =* 7.2 Hz, 2H), 2.71 (t, *J =* 6.9 Hz, 2H), 2.53 (s, 3H), 2.09–2.00 (m, 2H). ^13^C NMR (75 MHz, CDCl_3_) *δ* 163.6, 152.5, 151.3, 150.4, 138.2, 135.9, 133.5, 133.0, 118.3, 117.8, 71.8, 60.7, 49.0, 40.0, 35.5, 34.3, 29.0, 28.0. HRMS (ESI^+^): calcd. for C_22_H_26_N_3_O_4_Br_4_ [M + H]^+^, 711.8657; found, 711.8660.


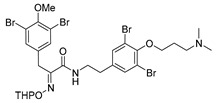


*(E)-N-[3,5-Dibromo-4-[3-(dimethylamino)propoxy]phenethyl]-3-(3,5-dibromo-4-methoxyphenyl)-2-[[(tetrahydro-2H-pyran-2-yl)oxy]imino]propanamide* (**27**)

A 20-mL Biotage MW tube was charged with purpurealidin E **25** [15,27] (0.17 g, 0.44 mmol), carboxylic acid **22a** (0.20 g, 0.44 mmol), EDC·HCl (0.13 g, 0.66 mmol, 1.5 equiv.), HOBt (0.10 g, 0.66 mmol, 1.2 equiv.), DIPEA (0.12 mL, 0.66 mmol, 1.2 equiv.) and dry DCM (15 mL). The tube was sealed and microwave irradiated at 60 °C for 5 h. The reaction mixture was diluted with DCM (10 mL) and washed with water (2 × 15 mL), a 1 M solution of HCl in H_2_O (15 mL) and brine (15 mL). The organic phase was dried over Na_2_SO_4_ (anhyd.), filtered and volatiles were removed in vacuo. The crude product was purified by column chromatography, Biotage SNAP Cartridge KP-Sil 25 g, isocratic elution: (DCM/MeOH, 7:3) to give **27** as a yellow oil (0.30 g, 83%). ^1^H NMR (400 MHz, CDCl_3_) *δ* 7.48 (s, 2H), 7.31 (s, 2H), 6.94 (t, *J* = 6.2 Hz, 1H), 5.36 (t, *J* = 2.8 Hz, 1H), 4.03 (t, *J* = 5.7 Hz, 2H), 3.89 (s, 1H), 3.82 (s, 3H), 3.58 (dd, *J* = 4.9, 8.1 Hz, 3H), 3.42 (m, 1H), 3.23–3.10 (m, 2H), 2.75 (t, *J* = 7.3 Hz, 2H), 2.69 (s, 6H), 2.29 (m, 2H), 1.89–1.59 (m, 6H). 


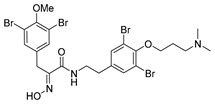


*(E)-N-[3,5-Dibromo-4-[3-(dimethylamino)propoxy]phenethyl]-3-(3,5-dibromo-4-methoxyphenyl)-2-(hydroxyimino)propanamide* (**29**)

THP ether **27** (0.30 g, 0.36 mmol) was dissolved to dry DCM (7 mL), and TFA (3 mL) was added under argon atmosphere. The reaction mixture was stirred at room temperature for 17 h. It was then diluted by adding DCM (10 mL) and washed with a 2 M solution of NaOH in H_2_O (15 mL) and water (15 mL) until pH was neutral. The aqueous phase was back extracted with DCM (10 mL) and the combined organic phase was dried over Na_2_SO_4_, filtered and concentrated in vacuo to give crude product (0.21 g, 80%). The crude product was purified by column chromatography, Biotage SNAP Cartridge KP-Sil 25 g, gradient elution: (DCM/MeOH, 2→20%) to give **29** as a yellow oil (0.074 g, 28%). ^1^H NMR (400 MHz, CD_3_OD) *δ* 7.48 (s, 2H), 7.43 (s, 2H), 4.02 (t, *J* = 5.9 Hz, 2H), 3.82 (s, 3H), 3.44 (dd, *J* = 6.7, 7.6 Hz, 2H), 3.35 (s, 2H), 3.15–2.98 (m, 2H), 2.85–2.69 (m, 2H), 2.64 (s, 6H), 2.15 (dq, *J* = 5.8, 7.7 Hz, 2H). ^13^C NMR (101 MHz, CD_3_OD) *δ* 165.4, 153.8, 152.5, 152.1, 140.0, 137.4, 134.5, 134.4, 118.8, 118.6, 71.8, 61.0, 57.3, 44.6, 41.4, 35.2, 28.8, 27.7. HRMS (ESI^+^): calcd. for C_23_H_27_Br_4_N_3_O_4_ [*M*+H]^+^ 725.8813, found: 725.8809.

##### General Method for the Amide Coupling

Carboxylic acid (0.20 g), aniline or amine (1–1.5 equiv.), EDC⋅HCl (1.5 equiv.), HOBt (1.5 equiv.), and DIPEA (1.5 equiv.) were dissolved in dry DCM (5 mL). The mixture was irradiated by microwaves for 2 h at 60 °C. The TLC indicated the completion of the reaction using vanillin as a visualization reagent. The reaction mixture was diluted with DCM (20 mL) and washed with water (2 × 15 mL) and a 2 M solution of HCl in H_2_O (2 × 15 mL). The organic layer was dried over anhyd. Na_2_SO_4_, filtered and concentrated in vacuo. The products were purified with flash column chromatography.


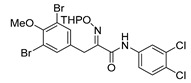


*(E)-3-(3,5-Dibromo-4-methoxyphenyl)-N-(3,4-dichlorophenyl)-2-[[(tetrahydro-2H-pyran-2-yl)oxy]-imino]propanamide* (**30-THP**)

Carboxylic acid **22a** (0.20 g, 0.44 mmol) and 3,4-dichloroaniline (0.11 g, 0.67 mmol, 1.5 equiv.) were reacted according to the general procedure for amide coupling. The product was purified by column chromatography, Biotage SNAP Cartridge KP-Sil 25 g and Ultra 25 g, gradient elution: (heptane/EtOAc, 12→100%) to give 30-THP as a crude product (0.19 g, 73%). ^1^H NMR (400 MHz, CDCl_3_) *δ* 8.64 (s, 1H), 7.87 (d, *J* = 1.9 Hz, 1H), 7.52 (s, 2H), 7.41–7.38 (m, 2H), 5.49 (t, *J* = 2.6 Hz, 1H), 3.99 (d, *J* = 13.1 Hz, 1H), 3.89 (d, *J* = 13.1 Hz, 1H), 3.84 (s, 3H), 3.65–3.59 (m, 2H), 1.93–1.59 (m, 6H).


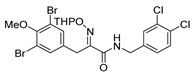


*(E)-3-(3,5-Dibromo-4-methoxyphenyl)-N-(3,4-dichlorobenzyl)-2-[[(tetrahydro-2H-pyran-2-yl)oxy]-imino]propanamide* (**31-THP**)

Carboxylic acid **22a** (0.20 g, 0.44 mmol) and 3,4-dichlorobenzylamine (0.090 mL, 0.67 mmol, 1.5 equiv.) were reacted according to the general procedure for amide coupling. The product was purified by column chromatography, Biotage SNAP Cartridge KP-Sil 25 g, gradient elution: (heptane/EtOAc, 7→60%) to give **31-THP** as a light yellow solid (0.23 g, 84%). ^1^H NMR (400 MHz, CDCl_3_) *δ* 7.50 (s, 2H), 7.39 (d, *J* = 8.2 Hz, 1H), 7.37 (d, *J* = 2.0 Hz, 1H), 7.19 (t, *J* = 5.9 Hz, 1H), 7.12 (dd, *J* = 2.1, 8.2 Hz, 1H), 5.38 (s, 1H), 4.54 (dd, *J* = 6.7, 15.2 Hz, 1H), 4.35 (dd, *J* = 5.8, 15.2 Hz, 1H), 3.95 (d, *J* = 13.1 Hz, 1H), 3.85 (d, 13.1 Hz, 1H), 3.84 (s, 1H), 3.61–3.55 (m, 2H), 1.87–1.56 (m, 6H).


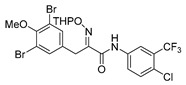


*(E)-N-[4-Chloro-3-(trifluoromethyl)phenyl]-3-(3,5-dibromo-4-methoxyphenyl)-2-[[(tetrahydro-2H-pyran-2-yl)oxy]imino]propanamide* (**32-THP**)

Carboxylic acid **22a** (0.19 g, 0.42 mmol) and 4-chloro-3-(trifluoromethyl)aniline (0.12 g, 0.63 mmol, 1.5 equiv.) were reacted according to the general procedure for amide coupling. The product was purified by column chromatography, Biotage SNAP Cartridge KP-Sil 25 g, gradient elution: (heptane/EtOAc, 7→60%). to give **32-THP** as an oil (0.19 g, 72%). ^1^H NMR (400 MHz, CDCl_3_) *δ* 8.73 (s, 1H), 7.95 (d, *J* = 2.6 Hz, 1H), 7.79 (dd, *J* = 2.6, 8.7 Hz, 1H), 7.52 (s, 2H), 7.46 (d, *J* = 8.8 Hz, 1H), 5.50 (t, *J* = 2.7 Hz, 1H), 4.00 (d, *J* = 13.2 Hz, 1H), 3.90 (d, *J* = 13.2 Hz, 1H), 3.84 (s, 3H), 3.66–3.59 (m, 2H), 1.95–1.66 (m, 6H).


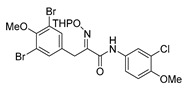


*(E)-N-(3-Chloro-4-methoxyphenyl)-3-(3,5-dibromo-4-methoxyphenyl)-2-[[(tetrahydro-2H-pyran-2-yl)oxy]imino]propanamide* (**33-THP**)

Carboxylic acid **22a (**0.20 g, 0.44 mmol) and 3-chloro-4-methoxyaniline (0.11 g, 0.67 mmol, 1.5 equiv.) were reacted according to the general procedure for amide coupling. The product was purified by column chromatography, Biotage SNAP Cartridge KP-Sil 25 g, gradient elution: (hexane/EtOAc, 7→60%) to give **33-THP** as a light yellow solid (0.20 g, 77%). ^1^H NMR (400 MHz, CDCl_3_) *δ* 8.51 (s, 1H), 7.70 (d, *J* = 2.6 Hz, 1H), 7.53 (s, 2H), 7.44 (dd, *J* = 2.6, 8.9 Hz, 1H), 6.89 (d, *J* = 8.9 Hz, 1H), 5.50–5.46 (m, 1H), 3.99 (d, *J* = 13.2 Hz, 1H), 3.89 (d, *J* = 13.2 Hz, 1H), 3.89 (s, 3H), 3.84 (s, 3H), 3.65–3.60 (m, 2H), 1.92–1.66 (m, 6H).


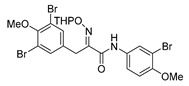


*(E)-N-(3-Bromo-4-methoxyphenyl)-3-(3,5-dibromo-4-methoxyphenyl)-2-[[(tetrahydro-2H-pyran-2-yl)oxy]imino]propanamide* (**34-THP**) 

Carboxylic acid **22a** (0.20 g, 0.44 mmol) and 3-bromo-4-methoxyaniline (0.13 g, 0.67 mmol, 1.5 equiv.) were reacted according to the general procedure for amide coupling. The product was purified by column chromatography, Biotage SNAP Cartridge KP-Sil 25 g, gradient elution: (heptane/EtOAc, 7→60%) to give **34-THP** as a light yellow solid (0.20 g, 71%). ^1^H NMR (400 MHz, CDCl_3_) *δ* 8.51 (s, 1H), 7.85 (d, *J* = 2.6 Hz, 1H), 7.53 (s, 2H), 7.51 (dd, *J* = 2.6, 8.9 Hz, 1H), 6.86 (d, *J* = 8.9 Hz, 1H), 5.47 (d, *J* = 2.9 Hz, 1H), 3.99 (d, *J* = 13.1 Hz, 1H), 3.92–3.87 (m, 4H), 3.84 (s, 3H), 3.65–3.60 (m, 2H), 1.91–1.66 (m, 6H).


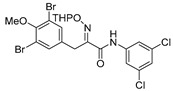


*(E)-3-(3,5-Dibromo-4-methoxyphenyl)-N-(3,5-dichlorophenyl)-2-[[(tetrahydro-2H-pyran-2-yl)oxy]-imino]propenamide* (**35-THP**)

Carboxylic acid **22a** (0.20 g, 0.44 mmol) and 3,5-dichloroaniline (0.11 g, 0.67 mmol, 1.5 equiv.) were reacted according to the general procedure for amide coupling. The product was purified by column chromatography, Biotage SNAP Cartridge KP-Sil 25 g and SNAP KP-NH 11g, gradient elution: (heptane/EtOAc, 7→60%). to give **35-THP** as a yellow oil (0.18 g, 69%). ^1^H NMR (400 MHz, CDCl_3_) *δ* 8.64 (s, 1H), 7.58 (d, *J* = 1.8 Hz, 2H), 7.52 (s, 2H), 7.12 (t, *J* = 1.8 Hz, 1H), 5.48 (t, *J* = 2.7 Hz, 1H), 3.98 (d, *J* = 13.2 Hz, 1H), 3.88 (d, *J* = 13.2 Hz, 1H) 3.84 (s, 3H), 3.65–3.59 (m, 2H), 1.92–1.66 (m, 6H).


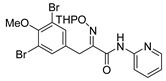


*(E)-3-(3,5-Dibromo-4-methoxyphenyl)-N-(pyridin-2-yl)-2-[[(tetrahydro-2H-pyran-2-yl)oxy]imino] propanamide* (**36-THP**)

Carboxylic acid **22a** (0.20 g, 0.44 mmol) and 2-aminopyridine (0.063 g, 0.67 mmol, 1.5 equiv.) were reacted according to the general procedure for amide coupling. The product was purified by column chromatography, Biotage SNAP Cartridge KP-Sil 25 g, gradient elution: (hexane/EtOAc, 12→60%) to give **36-THP** as a light yellow solid (0.11 g, 45%). ^1^H NMR (400 MHz, CDCl_3_) *δ* 9.26 (s, 1H), 8.32–8.28 (m, 1H), 8.24 (d, *J* = 8.4 Hz, 1H), 7.76–7.70 (m, 1H), 7.53 (s, 2H), 7.06 (ddd, *J* = 0.8, 4.9, 7.3 Hz, 1H), 5.47 (t, *J* = 2.9 Hz, 1H), 3.99 (d, *J =* 13.2 Hz, 1H), 3.89 (d, *J =* 13.2 Hz, 1H), 3.83 (s, 3H), 3.64–3,61 (m, 2H), 1.91–1.64 (m, 6H). ^13^C NMR (100 MHz, CDCl_3_) *δ* 160.5, 153.0, 151.8, 150.7, 148.1, 138.6, 134.6, 133.7, 120.2, 118.0, 114.2, 102.3, 62.5, 60.7, 28.8, 28.5, 25.1, 19.0. HRMS (ESI^+^): calculated 525.9977 (C_20_H_22_Br_2_N_3_O_4_), found 525.9972.


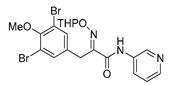


*(E)-3-(3,5-Dibromo-4-methoxyphenyl)-N-(pyridin-3-yl)-2-[[(tetrahydro-2H-pyran-2-yl)oxy]imino] propanamide* (**37-THP**)

Carboxylic acid **22a** (0.42 g, 0.94 mmol), 3-aminopyridine (0.11 g, 1.12 mmol, 1.2 equiv.) were reacted according to the general procedure for amide coupling. The product was purified by column chromatography Biotage SNAP Cartridge KP-Sil 25 g eluent (DCM/MeOH (0→10%) gradient to give the product **37-THP** as an oil (0.34 g, 68%). ^1^H NMR (400 MHz, CDCl_3_) *δ* 8.71 (d, *J* = 10.1 Hz, 1H), 8.38 (s, 1H), 8.20 (ddd, *J* = 1.4, 2.7, 8.4 Hz, 1H), 7.53 (s, 2H), 7.30 (dd, *J* = 4.6, 8.4 Hz, 1H), 5.51 (t, *J* = 2.9 Hz, 1H), 4.06–3.87 (m, 2H), 3.84 (s, 3H), 3.67–3.59 (m, 2H), 1.90–1.59 (m, 6H).


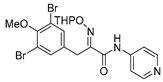


*(E)-3-(3,5-Dibromo-4-methoxyphenyl)-N-(pyridin-4-yl)-2-[[(tetrahydro-2H-pyran-2-yl)oxy]imino] propanamide* (**38-THP**)

Carboxylic acid **22a** (0.20 g, 0.45 mmol) and 4-aminopyridine (0.040 g, 0.45 mmol) were reacted according to the general procedure for amide coupling. The product was purified by column chromatography Biotage SNAP Cartridge KP-Sil 10 g, eluent (DCM/MeOH, 0→10%) gradient to give the product **38-THP** as an oil (0.049 g, 21%). ^1^H NMR (400 MHz, CDCl_3_) *δ* 8.55 (d, *J* = 5.6 Hz, 2H), 7.64 (d, *J* = 5.8 Hz, 2H), 7.52 (s, 2H), 5.51 (t, 1H), 4.04–3.86 (m, 2H), 3.84 (s, 3H), 3.66–3.59 (m, 2H), 1.85–1.60 (m, 6H).


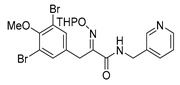


*(E)-3-(3,5-Dibromo-4-methoxyphenyl)-N-(pyridin-3-ylmethyl)-2-[[(tetrahydro-2H-pyran-2-yl)oxy]imino) propanamide* (**39-THP**)

Carboxylic acid **22a** (0.20 g, 0.44 mmol) and 3-picolylamine (0.068 mL, 0.67 mmol, 1.5 equiv.) were reacted according to the general procedure for amide coupling. The product was purified by column chromatography, Biotage SNAP Cartridge KP-Sil 25 g, gradient elution: (DCM/MeOH, 0→10%) to give **39-THP** as an oil (0.20 g, 82%). ^1^H NMR (400 MHz, CDCl_3_) *δ* 8.55 (br, 2H), 7.63 (d, *J* = 7.8 Hz, 1H), 7.51 (s, 2H), 7.30–7.25 (m, 1H), 7.22 (t, *J* = 6.1 Hz, 1H), 5.36 (t, *J* = 2.4 Hz, 1H), 4.61 (dd, *J* = 6.6, 15.1 Hz, 1H), 4.44 (dd, *J* = 5.8, 15.1 Hz, 1H), 3.94 (d, *J* = 13.2 Hz, 1H), 3.88–3.81 (m, 4H), 3.60–3.55 (m, 2H), 1.88–1.62 (m, 6H).


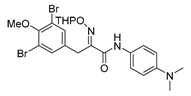


*(E)-3-(3,5-Dibromo-4-methoxyphenyl)-N-(4-(dimethylamino)phenyl)-2-[[(tetrahydro-2H-pyran-2-yl)oxy]imino]propanamide* (**40-THP**)

Carboxylic acid **22a** (0.22 g, 0.49 mmol and 4-(dimethylamino)aniline (0.060 g, 0.49 mmol) were reacted according to the general procedure for amide coupling. The product was purified by column chromatography, Biotage SNAP Cartridge KP-Sil 25 g, gradient elution: (EtOAc/MeOH, 0→20%) to give **40-THP** as an oil (0.16 g, 56%). ^1^H NMR (400 MHz, CDCl_3_) *δ* 7.55 (s, 2H), 7.45 (d, *J* = 9.0 Hz, 2H), 6.72 (d, *J* = 8.5 Hz, 2H), 5.47 (t, 1H), 4.03-3.89 (m, 2H), 3.83 (s, 3H), 3.65–3.60 (m, 2H), 2.93 (s, 6H), 1.91–1.57 (m, 6H)


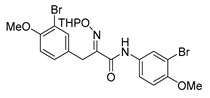


*(E)-N,3-Bis(3-bromo-4-methoxyphenyl)-2-[[(tetrahydro-2H-pyran-2-yl)oxy]imino]propenamide* (**41-THP**)

Carboxylic acid **22b** (0.40 g, 1.07 mmol) and 3-bromo-4-methoxyaniline (0.12 g, 1.29 mmol, 1.2 equiv.) were reacted according to the general procedure for amide coupling. The product was purified by column chromatography Biotage SNAP Cartridge KP-Sil 25 g eluent (DCM/MeOH (0→10%) gradient to give **41-THP** as an oil (0.43 g, 72%). ^1^H NMR (400 MHz, CDCl_3_) *δ* 8.54 (s, 1H), 7.86 (d, *J* = 2.6 Hz, 1H), 7.58 (d, *J* = 2.2 Hz, 1H), 7.49 (dd, *J* = 2.6, 8.9 Hz, 1H), 7.29 (dd, *J* = 2.2, 8.4 Hz, 1H), 6.85 (d, *J* = 8.9 Hz, 1H), 6.80 (d, *J* = 8.5 Hz, 1H), 5.46 (t, *J* = 2.8 Hz, 1H), 3.99 (d, *J* = 13.0 Hz, 1H), 3.91 (d, *J* = 13.0 Hz, 1H), 3.87 (s, 3H), 3.85 (s, 3H), 3.71–3.58 (m, 2H), 1.92–1.60 (m, 6H).


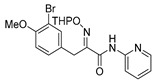


*(E)-3-(3-Bromo-4-methoxyphenyl)-N-(pyridin-2-yl)-2-[[(tetrahydro-2H-pyran-2-yl)oxy]imino]propanamide* (**42-THP**)

Carboxylic acid **22b** (0.40 g, 1.07 mmol) and 2-aminopyridine (0.12 g, 1.29 mmol, 1.2 equiv.) were reacted according to the general procedure for amide coupling. The product was purified by column chromatography Biotage SNAP Cartridge KP-Sil 25 g eluent (DCM/MeOH (0→10%) gradient to give **42-THP** as an oil (0.31 g, 64%). ^1^H NMR (400 MHz, CDCl_3_) *δ* 9.27 (s, 1H), 8.29 (ddd, *J* = 0.9, 2.0, 4.9 Hz, 1H), 8.23 (dt, *J* = 1.0, 8.4 Hz, 1H), 7.70 (ddd, *J* = 1.9, 7.4, 8.5 Hz, 1H), 7.57 (d, *J* = 2.2 Hz, 1H), 7.07–7.01 (m, 1H), 6.80 (d, *J* = 8.4 Hz, 1H), 5.47–5.44 (m, 1H), 3.99 (d, *J* = 13.1 Hz, 1H), 3.91 (d, *J* = 13.1 Hz, 1H), 3.84 (s, 3H), 3.68–3.60 (m, 2H), 1.90–-1.61 (m, 6H).


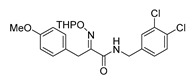


*(E)-N-(3,4-Dichlorobenzyl)-3-(4-methoxyphenyl)-2-[[(tetrahydro-2H-pyran-2-yl)oxy]imino]propanamide* (**43-THP**)

Carboxylic acid **22d** (0.40 g, 1.36 mmol) and 3,4-dichlorobenzylamine (0.11 g, 1.12 mmol, 1.2 equiv.) were reacted according to the general procedure for amide coupling. **43-THP** was obtained as light brown solid (0.54 g, 87%). ^1^H NMR (400 MHz CDCl_3_) *δ* 7.37 (d, *J* = 8.2 Hz, 1H), 7.34 (d, *J* = 2.1 Hz, 1H), 7.24 (s, 1H), 7.11–7.06 (m, 1H), 6.83–6.79 (m, 2H), 5.38–5.35 (m, 1H), 4.52 (dd, *J* = 6.7, 15.3 Hz, 1H), 4.34 (dd, *J* = 5.8, 15.2 Hz, 1H), 3.95 (d, *J* = 13.1 Hz, 1H), 3.85 (d, *J* = 13.1 Hz, 1H), 3.77 (s, 3H), 3.69–3.55 (m, 2H), 1.88–1.54 (m, 6H).


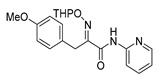


*(E)-3-(4-Methoxyphenyl)-N-(pyridin-2-yl)-2-[[(tetrahydro-2H-pyran-2-yl)oxy]imino]propanamide* (**44-THP**)

Carboxylic acid **22d** (0.40 g, 1.37 mmol), 2-aminopyridine (0.13 g, 1.37 mmol) were reacted according to the general procedure for amide coupling. The product was purified by column chromatography Biotage SNAP Cartridge KP-Sil 25 g and 10 g, eluent (EtOAc/MeOH, 0→10%) gradient to give **44-THP** as an oil (0.095 g, 19%). ^1^H NMR (400 MHz, CD_3_OD) *δ* 8.28 (ddd, *J* = 5.0, 1.9, 0.9 Hz, 1H), 8.18 (dt, *J* = 8.4, 1.0 Hz, 1H), 7.81 (ddd, *J* = 8.4, 7.4, 1.9 Hz, 1H), 7.25 (d, *J* = 8.8 Hz, 2H), 7.13 (ddd, *J* = 7.4, 5.0, 1.0 Hz, 1H), 6.83 (d, *J* = 8.8 Hz, 2H), 3.96-3.79 (m, 2H), 3.73 (s, 3H), 3.66–3.48 (m, 2H), 1.91–1.46 (m, 6H).


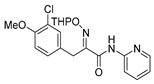


*(E)-3-(3-Chloro-4-methoxyphenyl)-N-(pyridin-2-yl)-2-[[(tetrahydro-2H-pyran-2-yl)oxy]imino]propanamide* (**45-THP**)

Carboxylic acid **22c** (0.32 g, 0.98 mmol) and 2-aminopyridine (0.093 g, 0.98 mmol) were reacted according to the general procedure for amide coupling. The product was purified by column chromatography Biotage SNAP Cartridge KP-Sil 10 g, eluent (DCM/MeOH, 0→10%) gradient to give **45-THP** as an oil (0.29 g, 74%). ^1^H NMR (400 MHz, CDCl_3_) *δ* 8.28 (ddd, *J* = 4.9, 1.9, 0.9 Hz, 1H), 8.24 (dt, *J* = 8.4, 1.0 Hz, 1H), 7.71 (ddd, *J* = 8.4, 7.4, 1.9 Hz, 1H), 7.40 (d, *J* = 2.2 Hz, 1H), 7.22 (dd, *J* = 8.4, 2.2 Hz, 1H), 7.04 (ddd, *J* = 7.4, 4.9, 1.0 Hz, 1H), 6.82 (d, *J* = 8.5 Hz, 1H), 5.46 (t, 1H), 3.99 (d, *J* = 13.1 Hz, 1H), 3.91 (d, *J* = 13.1 Hz, 1H), 3.85 (s, 3H), 3.68–3.56 (m, 2H), 1.90–1.57 (m, 6H).

##### General Method for THP Deprotection

THP ethers **30-THP**–**45-THP** (0.16 g, 0.27 mmol) and TFA (3 mL) were dissolved in dry DCM (7 mL). The reaction mixture was stirred under argon atmosphere for 3 d. Subsequently, the reaction mixture was quenched with a 2 M solution of NaOH in H_2_O (15 mL) and extracted with DCM (2 × 15 mL). The combined organic layers were dried over anhyd. Na_2_SO_4_, filtered and concentrated in vacuo. The crude products were purified using flash column chromatography.


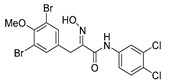


*(E)-3-(3,5-Dibromo-4-methoxyphenyl)-N-(3,4-dichlorophenyl)-2-(hydroxyimino)propanamide* (**30**)

The general procedure for THP deprotection was used, starting from ether **30** (0.16 g, 0.27 mmol). The worked-up reaction mixture was attempted to be purified using column chromatography Biotage SNAP Cartridge KP-Sil 25 g and SNAP Ultra 10 g without success. A successful purification was achieved by using Biotage SNAP Cartridge KP-NH 11 g, gradient elution: (heptane/EtOAc, 12→100%). The obtained oil was recrystallized from CHCl_3_ to give **30** as a white solid (0.052 g, 36%). Mp: 198 °C (decomposed).^1^H NMR (400 MHz, *d*_6_-DMSO) *δ* 12.38 (s, 1H), 10.30 (s, 1H), 8.08 (d, *J* = 2.4 Hz, 1H), 7.71 (dd, *J* = 2.5, 8.9 Hz, 1H), 7.57 (d, *J* = 8.8 Hz, 1H), 7.50 (s, 2H), 3.86 (s, 2H), 3.76 (s, 3H). ^13^C NMR (101 MHz, *d*_6_-DMSO) *δ* 162.2, 151.9, 151.3, 138.4, 136.0, 132.9, 130.8, 130.5, 125.3, 121.4, 120.3, 117.2, 60.4, 27.9. HRMS (ESI^+^): calculated 508.8670 (C_16_H_13_Br_2_Cl_2_N_2_O_3_), found 508.8670.


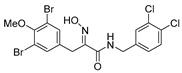


*(E)-3-(3,5-Dibromo-4-methoxyphenyl)-N-(3,4-dichlorobenzyl)-2-(hydroxyimino)propanamide* (**31**)

Unlike in the general procedure, compound **31-THP** (0.20 g, 0.33 mmol) was deprotected using a 2 M solution of HCl in Et_2_O (5 mL) in dry DCM (5 mL) under various conditions (sealed tube, 2 h, 60 °C, MW; sealed tube, 2 h, 70 °C, oil bath; sealed tube, 12 h, 30 °C, oil bath; reflux under argon, 60 h). The worked-up reaction mixture was purified twice by column chromatography, using Biotage SNAP KP-Sil 25 g, gradient elution: (heptane/EtOAc, 12→100%) to give **31** as a light yellow solid (0.038 g, 22%). ^1^H NMR (400 MHz, CDCl_3_) *δ* 7.79 (s, 1H), 7.49 (s, 2H), 7.39 (d, *J* = 8.2 Hz, 1H), 7.35 (d, *J* = 2.0 Hz, 1H), 7.10 (dd, *J* = 2.0, 8.2 Hz, 1H), 7.02 (t, *J* = 5.5 Hz, 1H), 4.45 (d, *J* = 6.2 Hz, 2H), 3.90 (s, 2H), 3.85 (s, 3H). ^13^C NMR (101 MHz, CDCl_3_) *δ* 162.5, 153.0, 152.6, 138.1, 134.6, 133.6, 132.9, 131.9, 130.9, 129.7, 127.1, 118.1, 60.7, 42.6, 28.1. HRMS (ESI^+^): calculated 522.8825 (C_17_H_15_Br_2_Cl_2_N_2_O_3_), found 522.8827.


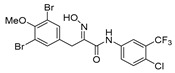


*(E)-N-[4-Chloro-3-(trifluoromethyl)phenyl]-3-(3,5-dibromo-4-methoxyphenyl)-2-(hydroxyimino) propanamide* (**32**)

The general procedure for THP deprotection was employed to deprotect **32-THP** (0.19 g, 0.30 mmol). The worked-up reaction mixture was purified by column chromatography, Biotage SNAP Cartridge KP-NH 11 g, gradient elution: (heptane/EtOAc, 12→100%) to give **32** as a light yellow solid (0.055 g, 33%). ^1^H NMR (400 MHz, CDCl_3_) *δ* 8.63 (s, 1H), 8.13 (s, 1H), 7.90 (d, *J* = 2.6 Hz, 1H), 7.77 (dd, *J* = 2.6, 8.7 Hz, 1H), 7.51 (s, 2H), 7.46 (d, *J* = 8.7 Hz, 1H), 3.95 (s, 2H), 3.84 (s, 3H). ^13^C NMR (101 MHz, CDCl3) δ 160.2, 153.0, 152.4, 135.9, 134.1, 133.5, 132.1, 129.1, 128.8, 127.2 (m), 123.6, 118.7 (q, *J*_CF_ = 5.7), 118.0, 60.6, 27.7. HRMS (ESI^+^): calculated 542.8934 (C_17_H_13_Br_2_ClF_3_N_2_O_3_), found 542.8937.


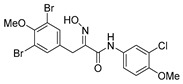


*(E)-N-(3-Chloro-4-methoxyphenyl)-3-(3,5-dibromo-4-methoxyphenyl)-2-(hydroxyimino)propanamide* (**33**)

The general procedure for THP deprotection was used, starting from ether **33-THP** (0.20 g, 0.34 mmol). The product was purified by column chromatography, Biotage SNAP Cartridge KP-NH 11 g, gradient elution: (cyclohexane/EtOAc, 12→100%) to give **33** as a light orange solid (0.086 g, 50%). ^1^H NMR (400 MHz, CDCl_3_) *δ* 8.41 (s, 1H), 8.07 (s, 1H), 7.64 (d, *J* = 2.6 Hz, 1H), 7.52 (s, 2H), 7.42 (dd, *J* = 2.6, 8.9 Hz, 1H), 6.88 (d, *J* = 8.9 Hz, 1H), 3.94 (s, 2H), 3.88 (s, 3H), 3.84 (s, 3H). ^13^C NMR (101 MHz, CDCl_3_) *δ* 160.0, 153.0, 152.7, 152.2, 134.5, 133.6, 130.8, 122.8, 122.4, 119.6, 118.1, 112.4, 60.7, 56.6, 27.9. HRMS (ESI^+^): calculated 504.9165 (C_17_H_16_Br_2_ClN_2_O_4_), found 504.9164.


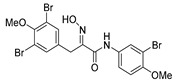


*(E)-N-(3-Bromo-4-methoxyphenyl)-3-(3,5-dibromo-4-methoxyphenyl)-2-(hydroxyimino)propanamide* (**34**)

The general procedure for THP deprotection was used, starting from ether **34**-THP (0.20 g, 0.31 mmol). The worked-up reaction mixture was attempted to be purified using Biotage SNAP Cartridge KP-Sil 25 g and SNAP Ultra 10 g. A successful purification was achieved by column chromatography using Biotage SNAP Cartridge KP-NH 11 g, gradient elution: (heptane/EtOAc, 12→100%) to give **34** as a yellow oily solid (0.087 g, 50%). ^1^H NMR (400 MHz, CDCl_3_) *δ* 8.45 (s, 1H), 8.42 (s, 1H), 7.78 (d, *J* = 2.6 Hz, 1H), 7.52 (s, 2H), 7.48 (dd, *J* = 2.6, 8.9 Hz, 1H), 6.85 (d, *J* = 8.9 Hz, 1H), 3.94 (s, 2H), 3.86 (s, 3H), 3.83 (s, 3H). ^13^C NMR (101 MHz, CDCl_3_) *δ* 160.1, 153.2, 152.9, 152.6, 134.6, 133.6, 131.1, 125.4, 120.4, 118.1, 112.2, 111.8, 60.7, 56.6, 27.9. HRMS (ESI^+^): calculated 548.8660 (C_17_H_16_Br_3_N_2_O_4_), found 548.8660.


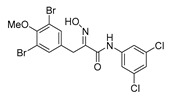


*(E)-3-(3,5-Dibromo-4-methxyphenyl)-N-(3,5-dichlorophenyl)-2-(hydroxyimino)propanamide* (**35**)

The general procedure for THP deprotection was used, starting from ether **35-THP** (0.18 g, 0.30 mmol). The worked-up reaction mixture was purified by column chromatography, Biotage SNAP Cartridge KP-NH 11 g, gradient elution: (heptane/EtOAc, 12→100%) to give **35** as a white solid (0.021 mg, 14%). ^1^H NMR (400 MHz, CD_3_OD) *δ* 7.71 (d, *J* = 1.8 Hz, 2H), 7.53 (s, 2H), 7.14 (t, *J* = 1.9 Hz, 1H), 3.90 (s, 2H), 3.81 (s, 3H). ^13^C NMR (101 MHz, CD_3_OD) *δ* 162.2, 152.6, 150.9, 140.2, 135.7, 134.7, 133.2, 123.2, 118.0, 117.3, 59.6, 27.3. HRMS (ESI^+^): calculated 508.8670 (C_16_H_13_Br_2_Cl_2_N_2_O_3_), found 508.8659.


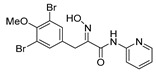


*(E)-3-(3,5-Dibromo-4-methoxyphenyl)-2-(hydroxyimino)-N-(pyridin-2-yl)propanamide* (**36**)

The general procedure for THP deprotection was used, starting from ether **36-THP** (0.087 g, 0.17 mmol). The product was purified by column chromatography, Biotage SNAP Cartridge KP-NH 11 g, gradient elution: (hexane/EtOAc, 12→100%). The obtained **36** as a white powder was further re-crystallized from CHCl_3_ for the X-ray to give transparent crystals (0.037 g, 51%). M.p.: 192–196 °C (decomposed). ^1^H NMR (400 MHz, CDCl_3_) *δ* 13.85 (s, 1H), 9.41 (s, 1H), 8.39 (d, *J* = 8.5 Hz, 1H), 8.32–8.27 (m, 1H), 7.89–7.84 (m, 1H), 7.57 (s, 2H), 7.19 (ddd, *J* = 0.8, 5.2, 7.3 Hz, 1H), 4.02 (s, 2H), 3.84 (s, 3H. ^13^C NMR (101 MHz, CDCl_3_) *δ* 161.7, 152.8, 151.7, 150.1, 146.4, 140.2, 135.2, 133.7, 120.4, 118.0, 115.7, 60.7, 28.2. HRMS (ESI^+^): calculated 441.9402 (C_15_H_14_Br_2_N_3_O_3_), found 441.9401. LC-MS: [M + H]^+^ *m/z* 442 (*t_R_* = 5.39 min), >99%.


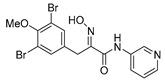


*(E)-3-(3,5-Dibromo-4-methoxyphenyl)-2-(hydroxyimino)-N-(pyridin-3-yl)propanamide* (**37**)

The general procedure for THP deprotection was used, starting from ether **37-THP** (0.34 g, 0.64 mmol). The product was purified by column chromatography, Biotage SNAP Cartridge KP-Sil 25 g, gradient elution: (hexane/acetone, 0→100%) to give **37** as a pale yellow solid (0.052 g, 18%). ^1^H NMR (400 MHz, CD_3_OD) *δ* 8.84 (dd, *J* = 2.5, 19.4 Hz, 1H), 8.30–8.16 (m, 2H), 7.53 (d, *J* = 8.4 Hz, 2H), 7.44–7.35 (m, 1H), 3.92 (s, 2H), 3.80 (s, 3H). ^13^C NMR (101 MHz, CD_3_OD) *δ* 163.8, 154.4, 153.9, 152.3, 145.3, 142.3, 137.1, 134.44, 129.41, 125.2, 118.7, 61.0, 28.7. HRMS (ESI^+^): calculated 441.9402 (C_15_H_14_N_3_O_3_Br_2_), found 441.9402.


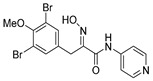


*(E)-*3-(3,5-Dibromo-4-methoxyphenyl)*2-(hydroxyimino)-N-(pyridin-4-yl)propanamide* (**38**)

The general procedure for THP deprotection was used, starting from ether **38-THP** (0.49 g, 0.093 mmol). The product was purified by column chromatography, Biotage SNAP Cartridge KP-Sil 10 g, gradient elution: (DCM/MeOH, 0→10%) to give **38** as a brownish solid (0.0099 g, 24%). ^1^H NMR (400 MHz, *d*_6_-acetone) *δ* 8.47 (s, 2H), 7.73 (d, *J* = 5.5 Hz, 2H), 7.59 (s, 2H), 3.98 (s, 2H), 3.82 (s, 3H). ^13^C NMR (101 MHz, *d*_6_-acetone) *δ* 162.9, 153.5, 152.2, 151.3, 145.8, 136.7, 134.3, 118.3, 114.5, 60.9, 28.4. HRMS (ESI^+^): calculated 441.9402 (C_15_H_14_N_3_O_3_Br_2_), found 441.9400.


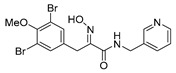


*(E)-3-(3,5-Dibromo-4-methoxyphenyl)-2-(hydroxyimino)-N-(pyridin-3-ylmethyl)propanamide* (**39**)

The general procedure for THP deprotection was used, starting from ether **39-THP** (0.17 g, 0.32 mmol). The product was purified by column chromatography, Biotage SNAP Cartridge KP-NH 11 g, gradient elution: (DCM/MeOH, 0→10%) to give **39** as a yellowish solid (0.039 g, 26%). ^1^H NMR (400 MHz, CDCl_3_) *δ* 8.52 (br s, 2H), 7.75 (d, *J* = 7.8 Hz, 1H), 7.52 (s, 2H), 7.39–7.33 (m, 1H), 7.16 (t, *J* = 6.1 Hz, 1H), 4.54 (d, *J* = 6.1 Hz, 2H), 3.91 (s, 2H), 3.84 (s, 3H). ^13^C NMR (101 MHz, CDCl_3_) *δ* 163.3, 152.7, 151.1, 148.0, 147.8, 137.5, 135.4, 134.7, 133.6, 124.4, 118.0, 60.7, 41.0, 28.2. HRMS (ESI^+^): calculated 455.9558 (C_16_H_16_Br_2_N_3_O_3_), found 455.9563.


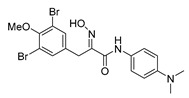


*(E)-3-(3,5-Dibromo-4-methoxyphenyl)-N-[4-(dimethylamino)phenyl]-2-(hydroxyimino)propanamide* (**40**)

The general procedure for THP deprotection was used, starting from ether **40-THP** (0.16 g, 0.27 mmol). The product was purified by column chromatography, Biotage SNAP Cartridge KP-Sil 25 g, gradient elution: (hexane/EtOAc, 7→60%). The obtained product was further re-crystallized from hexane and acetone to give **40** as a yellow solid (0.07 g, 50%). ^1^H NMR (400 MHz, *d*_6_-acetone) *δ* 7.61 (s, 2H), 7.56 (d, *J* = 9.1 Hz, 2H), 6.72 (d, *J* = 9.2 Hz, 2H), 3.96 (s, 2H), 3.82 (s, 3H), 2.90 (s, 6H). ^13^C NMR (101 MHz, *d*_6_-acetone) *δ* 161.2, 153.4, 153.0, 148.8, 137.2, 134.4, 129.1, 122.0, 118.2, 113.5, 60.9, 40.9, 28.5. HRMS (ESI^+^): calculated 483.9871 (C_18_H_20_N_3_O_3_Br_2_), found 483.9876.


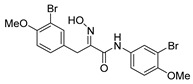


*(E)-N,3-Bis(3-bromo-4-methoxyphenyl)-2-(hydroxyimino)propanamide* (**41**)

The general procedure for THP deprotection was used, starting from ether **41-THP** (0.43 g, 0.78 mmol). The product was purified by column chromatography, Biotage SNAP Cartridge KP-Sil 25 g, gradient elution: (hexane/EtOAc, 0→60%) to give **41** as a yellow solid (0.087 g, 24%). ^1^H NMR (400 MHz, CD_3_OD) *δ* 7.85 (d, *J* = 2.5 Hz, 1H), 7.51–7.45 (m, 2H), 7.24 (dd, *J* = 2.2, 8.5 Hz, 1H), 6.92 (d, *J* = 8.9 Hz, 1H), 6.86 (d, *J* = 8.5 Hz, 1H), 3.88 (s, 2H), 3.81 (s, 3H), 3.78 (s, 3H). ^13^C NMR (101 MHz, CD_3_OD) *δ* 163.4, 155.9, 154.1, 153.3, 134.8, 133.0, 131.6, 130.5, 126.6, 121.9, 113.11, 113.09, 112.2, 112.1, 56.8, 56.6, 28.7. HRMS (ESI^+^): calculated 470.9555 (C_17_H_17_N_2_O_4_Br_2_), found 470.9554.


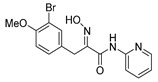


*(E)-3-(3-Bromo-4-methoxyphenyl)-2-(hydroxyimino)-N-(pyridin-2-yl)propanamide* (**42**)

The general procedure for THP deprotection was used, starting from ether **42-THP** (0.31 g, 0.68 mmol). The product was purified by column chromatography, Biotage SNAP Cartridge KP-Sil 25 g, gradient elution: (DCM/MeOH, 0→10%). The obtained solid was further re-crystallized from hexane to give **42** as a white solid (0.062 g, 25%). M.p.: 214–7 °C (decomposed). ^1^H NMR (400 MHz, *d*_6_-DMSO) *δ* 12.47 (s, 1H), 9.55 (s, 1H), 8.33 (ddd, *J* = 4.9, 1.9, 0.9 Hz, 1H), 8.07 (dt, *J* = 8.3, 1.0 Hz, 1H), 7.83 (ddd, *J* = 8.5, 7.3, 1.9 Hz, 1H), 7.45 (d, *J* = 2.1 Hz, 1H), 7.23 (dd, *J* = 8.5, 2.2 Hz, 1H), 7.16 (ddd, *J* = 7.3, 4.9, 1.0 Hz, 1H), 7.03 (d, *J* = 8.5 Hz, 1H), 3.84 (s, 2H), 3.80 (s, 3H). ^13^C NMR (101 MHz, *d*_6_-DMSO) *δ* 161.6, 153.9, 151.1, 150.6, 148.3, 138.5, 133.0, 130.0, 129.3, 120.0, 113.4, 112.7, 110.3, 56.2, 27.4. HRMS (ESI^+^): calculated 364.0297 (C_15_H_15_N_3_O_3_Br), found 364.0299.


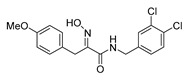


*(E)-N-(3,4-Dichlorobenzyl)-2-(hydroxyimino)-3-(4-methoxyphenyl)propanamide* (**43**)

The general procedure for THP deprotection was used, starting from ether **43-THP** (0.53 g, 1.18 mmol). The product was purified by column chromatography, Biotage SNAP Cartridge KP-Sil 25 g, gradient elution: (heptane/EtOAc, 0→100%) to give **43** as a yellow solid (0.057 g, 13%). ^1^H NMR (400 MHz, CD_3_OD) *δ* 7.39 (d, *J* = 8.3 Hz, 1H), 7.35 (d, *J* = 2.0 Hz, 1H), 7.22–7.15 (m, 2H), 7.13–7.07 (m, 1H), 6.82–6.72 (m, 2H), 4.36 (s, 2H), 3.87 (s, 2H), 3.74 (s, 3H). ^13^C NMR (101 MHz, CD_3_OD) *δ* 166.3, 159.7, 153.9, 141.1, 133.2, 131.7, 131.5, 131.1, 130.3, 129.9, 128.1, 114.8, 55.6, 42.7, 29.2. HRMS (ESI^+^): calculated 367.0616 (C_17_H_17_N_2_O_3_Cl_2_), found 367.0615.


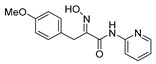


*(E)-2-(Hydroxyimino)-3-(4-methoxyphenyl)-N-(pyridin-2-yl)propanamide* (**44**)

The general procedure for THP deprotection was used, starting from ether **44-THP** (0.095 g, 0.26 mmol). The product was purified by column chromatography, Biotage SNAP Cartridge KP-Sil 10 g, gradient elution: (DCM/MeOH, 2→10%). The obtained product was further re-crystallized from hexane and acetone to give **44** as a white solid (0.02 g, 28%). ^1^H NMR (400 MHz, CD_3_OD) *δ* 8.26 (ddd, *J* = 5.0, 1.9, 0.9 Hz, 1H), 8.19 (dt, *J* = 8.4, 1.0 Hz, 1H), 7.81 (ddd, *J* = 8.4, 7.4, 1.9 Hz, 1H), 7.30–7.21 (m, 2H), 7.12 (ddd, J = 7.4, 5.0, 1.1 Hz, 1H), 6.85–6.76 (m, 2H), 3.92 (s, 2H), 3.74 (s, 3H). ^13^C NMR (101 MHz, CD_3_OD) *δ* 163.4, 159.7, 153.2, 152.3, 149.0, 140.0, 131.2, 129.9, 121.1, 115.1, 114.8, 55.6, 28.6. HRMS (ESI^+^): calculated 286.1192 (C_15_H_16_N_3_O_3_), found 286.1195.


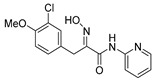


*(E)-3-(3-Chloro-4-methoxyphenyl)-2-(hydroxyimino)-N-(pyridin-2-yl)propanamide* (**45**)

The general procedure for THP deprotection was used, starting from ether **45-THP** (0.29 g, 0.72 mmol). The obtained product was further re-crystallized from acetone to give **45** as a white solid (0.05 g, 20%). M.p.: 218–219 °C (decomposed) ^1^H NMR (400 MHz, *d*_6_-acetone) *δ* 8.29 (ddd, *J* = 4.9, 1.9, 0.9 Hz, 1H), 8.20 (d, *J* = 8.3 Hz, 1H), 7.84–7.78 (m, 1H), 7.42–7.40 (m, 1H), 7.29 (ddt, *J* = 8.4, 2.2, 0.6 Hz, 1H), 7.12 (ddd, *J* = 7.4, 4.9, 1.0 Hz, 1H), 7.02 (d, *J* = 8.5 Hz, 1H), 3.96 (s, 2H), 3.85 (s, 3H). ^13^C NMR (101 MHz, *d*_6_-acetone) *δ* 162.0, 154.7, 152.8, 151.9, 149.2, 139.1, 131.5, 130.6, 129.7, 122.4, 120.7, 114.0, 113.3, 56.4, 28.2. HRMS (ESI^+^): calculated 320.0802 (C_15_H_15_N_3_O_3_Cl), found 320.0802.

### 4.2. Cell Lines

Human malignant melanoma cell line A-375 was kindly provided by Prof. Marikki Laiho (University of Helsinki, Finland) and Hs27 human skin fibroblast cell line was kindly provided by Dr. Carmen Escobedo-Lucea (University of Helsinki, Finland). The cells were maintained in Glutamax high glucose Dulbecco’s Modified Eagle’s Medium (DMEM, Gibco), supplemented with 10% fetal bovine serum (FBS, Gibco), at 37 °C, 5% CO_2_.

### 4.3. Analysis of Selectivity to Cancer Cells

The cells were seeded to white frame and clear bottom 96-well plates (Perkin Elmer) at the density of 10,000 cells/well for A-375 cell line and 7500 cells/well for Hs27 cell line. The cells were grown at 37 °C, 5% CO_2_ until they reached 70–80% confluence (approximately 24 h). Stock solutions of test compounds and a positive control (camptothecin, Sigma-Aldrich, Saint Louis, MO, USA) were prepared in DMSO and diluted into assay medium (growth medium with 5% FBS) to the final concentration. Final DMSO concentration was 0.5% in all samples. The culture medium was removed from the plate and compounds added, 200 µL/well. After 48-h incubation, the amount of ATP, which is directly proportional to the number of cells present in culture, was quantified using CellTiter-Glo^®^ Luminescent Cell Viability kit (Promega, Madison, WI, USA), according to manufacturer’s instructions.

Origin Graphing and Analysis, version 8.6 (OriginLab, Northampton, MA, USA) was used for determination of CC_50_ values. The cancer cell selectivity index (SI) was calculated as a ratio of CC50 values between Hs27 fibroblasts and A-375 melanoma cells.

## 5. Conclusions

Several syntheses of bromotyrosines have been reported but the synthesis of bromotyrosines with monomethylated tyramine part have not been reported before. The selective removal of the protective groups from the tyramine fragment before the coupling reaction is a challenging step in the total synthesis of purpurealidin I (**1**). We succeeded at this by using trifluoroacetyl protection. This route can be utilized further for the synthesis of additional bromotyrosine derivatives possessing the monomethylated tyramine fragment. The synthesized simplified analogs without the tyramine fragment retained the cytotoxic activity. The selectivity towards melanoma cell line was generally low. The highest selectivity (SI 4.1) was demonstrated in the case of pyridin-2-yl compound (**36**). This shows that the marine cytotoxic bromotyrosines are promising scaffolds for developing cytotoxic agents and the full understanding of the elements of their SAR is still in very early stage. Further optimization of simplified bromotyrosine derivatives is needed to attain high selectivity.

## Figures and Tables

**Table 1:**
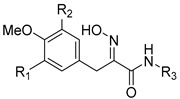
Structures of final hydroxyimino propanamides (**30**–**45**) from the Scheme 5. The corresponding THP-ethers (**23a**–**d**) are found in the Experimental part (Section 4.1.2) numbered as **30-THP**–**45-THP**.

Cmpd	R_1_	R_2_	R_3_	Cmpd	R_1_	R_2_	R_3_	Cmpd	R_1_	R_2_	R_3_
**30**	Br	Br	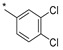	**35**	Br	Br	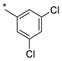	**40**	Br	Br	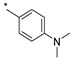
**31**	Br	Br	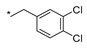	**36**	Br	Br	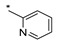	**41**	Br	H	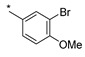
**32**	Br	Br	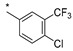	**37**	Br	Br	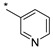	**42**	Br	H	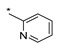
**33**	Br	Br	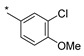	**38**	Br	Br	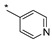	**43**	H	H	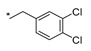
**34**	Br	Br	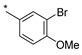	**39**	Br	Br	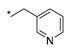	**44**	H	H	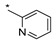
								**45**	Cl	H	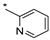

**Table 2:**
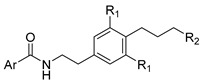
Structures of final amide compounds (**46**–**78**) [15].

Cmpd	Ar	R_1_	R_2_	Cmpd	Ar	R_1_	R_2_	Cmpd	Ar	R_1_	R_2_
**46**	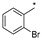	Br	N(CH_3_)_2_	**57**	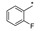	Br	N(CH_3_)_2_	**68**	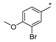	Br	N(CH_3_)_2_
**47**	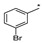	Br	N(CH_3_)_2_	**58**	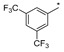	Br	N(CH_3_)_2_	**69**	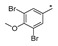	Br	N(CH_3_)_2_
**48**	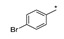	Br	N(CH_3_)_2_	**59**	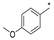	Br	N(CH_3_)_2_	**70**	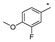	Br	N(CH_3_)_2_
**49**	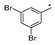	Br	C(CH_3_)_2_	**60**	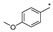	Br	NH(CH_3_)	**71**	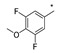	Br	N(CH_3_)_2_
**50**	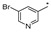	Br	N(CH_3_)_2_	**61**	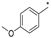	H	NH(CH_3_)	**72**	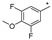	Br	NH(CH_3_)
**51**	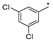	Br	N(CH_3_)_2_	**62**	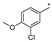	Br	N(CH_3_)_2_	**73**	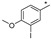	Br	N(CH_3_)_2_
**52**	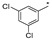	Br	C(CH_3_)_2_	**63**	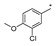	Br	NH(CH_3_)	**74**	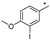	Br	NH(CH_3_)
**53**	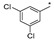	H	N(CH_3_)_2_	**64**	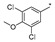	Br	N(CH_3_)_2_	**75**	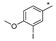	H	N(CH_3_)_2_
**54**	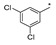	H	NH(CH_3_)	**65**	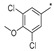	Br	NH(CH_3_)	**76**	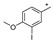	H	NH(CH_3_)
**55**	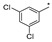	Br	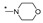	**66**	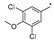	H	N(CH_3_)	**77**	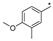	Br	N(CH_3_)_2_
**56**	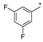	Br	N(CH_3_)_2_	**67**	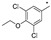	Br	N(CH_3_)_2_	**78**	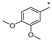	Br	N(CH_3_)_2_

**Table 3:** Cytotoxicity of purpurealidin I (**1**) and compounds (**29**–**78**) against human malignant melanoma cell line (A-375) and normal skin fibroblast cell line (Hs27). Camptothecin, a compound with high selectivity to cancer cells, was used as a positive control. The selectivity index of individual compounds was calculated as a ratio of CC_50_ in normal fibroblasts over CC_50_ in melanoma cells. CC_50_ = cytotoxic concentration that caused death of 50% cells. ND = not determined.

Compound	Primary Test Result (Cytotoxicity % at 50 µM in A-375 Cells)	CC_50_ (µM) in A-375 Cells	CC_50_ (µM) in Hs27 Cells	Selectivity Index
**1**	97.9	4.3	5.2	1.2
**29**	99.9	6.3	4.5	0.7
**30**	101.4	13.1	15.2	1.2
**31**	99.7	12.4	22.3	1.8
**32**	99.8	10.3	12.0	1.2
**33**	98.9	13.5	26.4	2.0
**34**	100.2	16.2	22.5	1.4
**35**	101.4	9.6	12.2	1.3
**36**	84.7	4.7	19.4	4.1
**37**	83.5	26.4	ND	ND
**38**	71.1	ND	ND	ND
**39**	93.2	22.1	ND	ND
**40**	99.1	27.8	ND	ND
**41**	99.2	19.8	ND	ND
**42**	2.7	ND	ND	ND
**43**	21.5	ND	ND	ND
**44**	0.4	ND	ND	ND
**45**	4.0	ND	ND	ND
**46**	59.2	ND	ND	ND
**47**	91.3	31.6	ND	ND
**48**	97.6	13.1	11.3	0.9
**49**	38.3	ND	ND	ND
**50**	65.0	ND	ND	ND
**51**	93.6	14.8	11.8	0.8
**52**	38.8	ND	ND	ND
**53**	90.0	27.7	ND	ND
**54**	101.4	20.4	ND	ND
**55**	31.3	ND	ND	ND
**56**	86.7	33.5	ND	ND
**57**	26.0	ND	ND	ND
**58**	97.4	13.2	6.8	0.5
**59**	39.6	ND	ND	ND
**60**	93.8	34.1	35.7	1.0
**61**	0.0	ND	ND	ND
**62**	96.5	17.7	ND	ND
**63**	99.9	11.0	11.7	1.1
**64**	98.4	12.3	11.8	1.0
**65**	99.7	6.5	7.2	1.1
**66**	98.6	15.4	ND	ND
**67**	97.0	7.7	7.2	0.9
**68**	96.8	14.8	14.9	1.0
**69**	97.9	13.4	7.5	0.6
**70**	45.3	ND	ND	ND
**71**	98.6	27.1	ND	ND
**72**	99.9	15.3	ND	ND
**73**	99.3	8.4	11.1	1.3
**74**	96.7	6.2	10.0	1.6
**75**	101.0	42.9	ND	ND
**76**	96.6	19.6	ND	ND
**77**	94.3	22.1	ND	ND
**78**	31.7	ND	ND	ND
Camptothecin	94.7	0.06	5.54	92.3

## Supplementary Materials

The following are available online at http://www.mdpi.com/1660-3397/16/12/481/s1, Appendix.pdf (synthesis, NMR spectra of compounds 1 and 36, and single crystal X-ray diffraction measurements), Compound36.cif and checkcif-Compound36.pdf
