# Supplementary material for: Synthesis and Antiproliferative Activity of Marine Bromotyrosine Purpurealidin I and Its Derivatives
Source: Mar Drugs. 2018 Dec 3;16(12):481. doi: 10.3390/md16120481 (PMC6316490; doi:10.3390/md16120481)

checkCIF/PLATON report

Structure factors have been supplied for datablock(s) shelx

THIS REPORT IS FOR GUIDANCE ONLY. IF USED AS PART OF A REVIEW PROCEDURE FOR PUBLICATION, IT SHOULD NOT REPLACE THE EXPERTISE OF AN EXPERIENCED CRYSTALLOGRAPHIC REFEREE.

No syntax errors found. CIF dictionary Interpreting this report

Datablock: shelx

```
Bond precision:  C-C = 0.0052 A          Wavelength=1.54184

Cell:            a=4.4118(3)    b=24.7517(14)     c=18.6721(11)
                 alpha=90       beta=96.690(6)     gamma=90
Temperature:     120 K

                Calculated                Reported
Volume          2025.1(2)                 2025.1(2)
Space group     P 21/n                    P 21/n
Hall group      -P 2yn                    -P 2yn
Moiety formula  C15 H13 Br2 N3 O3, C H Cl3 C15 H13 Br2 N3 O3, C H Cl3
Sum formula     C16 H14 Br2 Cl3 N3 O3     C16 H14 Br2 Cl3 N3 O3
Mr              562.45                    562.47
Dx,g cm-3       1.845                     1.845
Z               4                         4
Mu (mm-1)       8.928                     8.928
F000            1104.0                    1104.0
F000'           1105.08
h,k,lmax        5,28,21                   5,28,21
Nref            3342                      3240
Tmin,Tmax       0.552,0.585               0.176,1.000
Tmin'           0.038

Correction method= # Reported T Limits: Tmin=0.176 Tmax=1.000
AbsCorr = MULTI-SCAN

Data completeness= 0.969          Theta(max)= 63.989

R(reflections)= 0.0455( 2931)     wR2(reflections)= 0.1259( 3240)

S = 1.060                  Npar= 248
```

The following ALERTS were generated. Each ALERT has the format
test-name_ALERT_alert-type_alert-level.
Click on the hyperlinks for more details of the test.

Alert level C

```
THETM01_ALERT_3_C  The value of sine(theta_max)/wavelength is less than 0.590
            Calculated sin(theta_max)/wavelength =    0.5829
PLAT029_ALERT_3_C _diffrn_measured_fraction_theta_full value Low .      0.969 Note
PLAT790_ALERT_4_C Centre of Gravity not Within Unit Cell: Resd.  #          1 Note
              C15 H13 Br2 N3 O3
PLAT906_ALERT_3_C Large K value in the Analysis of Variance ......      2.466 Check
PLAT911_ALERT_3_C Missing # FCF Refl Between THmin & STh/L=  0.583        103 Report
```

Alert level G

```
PLAT002_ALERT_2_G Number of Distance or Angle Restraints on AtSite          3 Note
PLAT007_ALERT_5_G Number of Unrefined Donor-H Atoms .............           1 Report
PLAT172_ALERT_4_G The CIF-Embedded .res File Contains DFIX Records          1 Report
PLAT173_ALERT_4_G The CIF-Embedded .res File Contains DANG Records          1 Report
PLAT434_ALERT_2_G Short Inter HL..HL Contact Br2    ..  Cl1     ..       3.50 Ang.
PLAT790_ALERT_4_G Centre of Gravity not Within Unit Cell: Resd.  #          2 Note
              C H Cl3
PLAT860_ALERT_3_G Number of Least-Squares Restraints ............           2 Note
PLAT909_ALERT_3_G Percentage of Observed Data at Theta(Max) Still          85 % Note
PLAT933_ALERT_2_G Number of OMIT Records in Embedded .res File ...          2 Note
PLAT961_ALERT_5_G Dataset Contains no Negative Intensities .......     Please Check
PLAT978_ALERT_2_G Number C-C Bonds with Positive Residual Density.          1 Note
```

0 **ALERT level A** = Most likely a serious problem - resolve or explain
0 **ALERT level B** = A potentially serious problem, consider carefully
5 **ALERT level C** = Check. Ensure it is not caused by an omission or oversight
11 **ALERT level G** = General information/check it is not something unexpected

0 ALERT type 1 CIF construction/syntax error, inconsistent or missing data
4 ALERT type 2 Indicator that the structure model may be wrong or deficient
6 ALERT type 3 Indicator that the structure quality may be low
4 ALERT type 4 Improvement, methodology, query or suggestion
2 ALERT type 5 Informative message, check

It is advisable to attempt to resolve as many as possible of the alerts in all categories. Often the minor alerts point to easily fixed oversights, errors and omissions in your CIF or refinement strategy, so attention to these fine details can be worthwhile. In order to resolve some of the more serious problems it may be necessary to carry out additional measurements or structure refinements. However, the purpose of your study may justify the reported deviations and the more serious of these should normally be commented upon in the discussion or experimental section of a paper or in the "special_details" fields of the CIF. checkCIF was carefully designed to identify outliers and unusual parameters, but every test has its limitations and alerts that are not important in a particular case may appear. Conversely, the absence of alerts does not guarantee there are no aspects of the results needing attention. It is up to the individual to critically assess their own results and, if necessary, seek expert advice.

Publication of your CIF in IUCr journals

A basic structural check has been run on your CIF. These basic checks will be run on all CIFs submitted for publication in IUCr journals (*Acta Crystallographica*, *Journal of Applied Crystallography*, *Journal of Synchrotron Radiation*); however, if you intend to submit to *Acta Crystallographica Section C* or *E* or *IUCrData*, you should make sure that full publication checks are run on the final version of your CIF prior to submission.

Publication of your CIF in other journals

Please refer to the *Notes for Authors* of the relevant journal for any special instructions relating to CIF submission.

PLATON version of 27/03/2017; check.def file version of 24/03/2017

Datablock shelx - ellipsoid plot

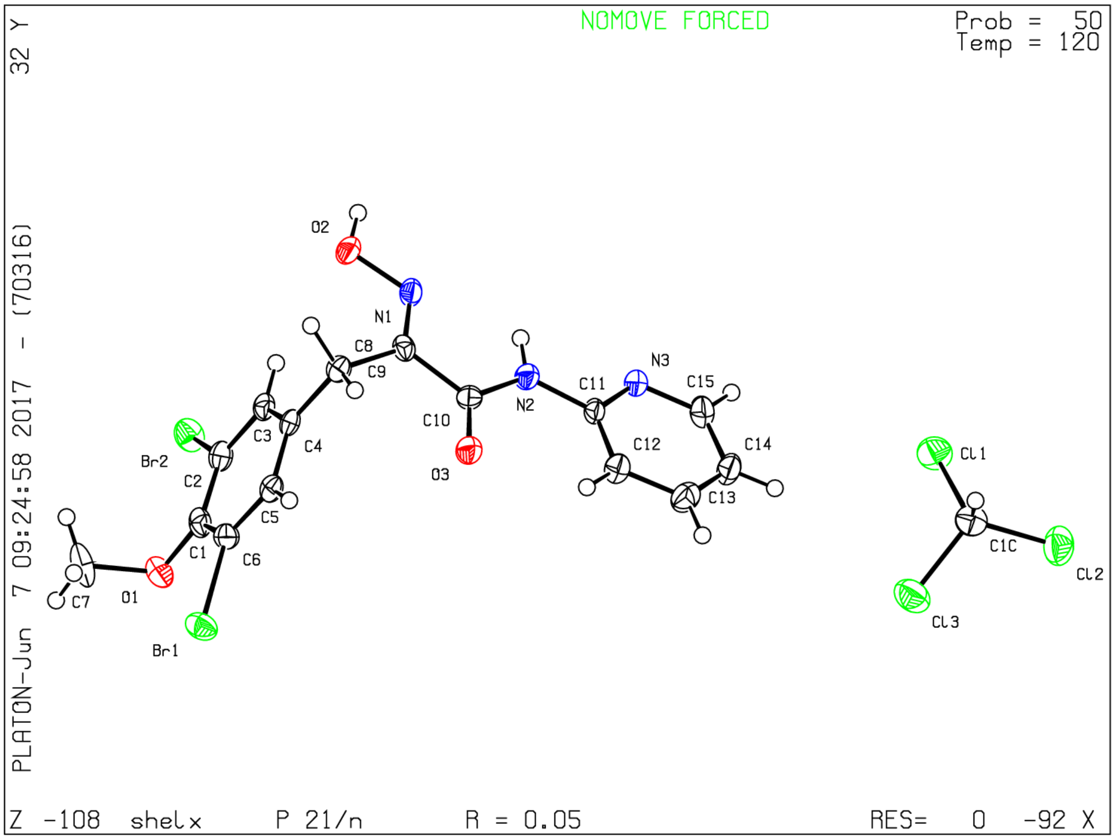

Supplement: Supplementary file 1 [file marinedrugs-16-00481-s001.zip › checkcif-Compound36.pdf]
