# Supplementary material for: Synthesis and Antiproliferative Activity of Marine Bromotyrosine Purpurealidin I and Its Derivatives
Source: Mar Drugs. 2018 Dec 3;16(12):481. doi: 10.3390/md16120481 (PMC6316490; doi:10.3390/md16120481)
Supplement: Supplementary file 1 [file marinedrugs-16-00481-s001.zip › marinedrugs-393678_Supporting Information.pdf]

Appendix Supporting Information

Synthesis and antiproliferative activity of marine bromotyrosine purpurealidin I and its derivatives

Chinmay Bhat[1†], Polina Ilina[2], Irene Tilli[1], Manuela Voráčová[1], Tanja Bruun[1], Victoria Barba[1], Nives Hribernik[1], Katja-Emilia Lillsunde[2], Eero Mäki-Lohiluoma[1], Tobias Rüffer[3], Heinrich Lang[3], Jari Yli-Kauhaluoma[1], Paula Kiuru[1], Päivi Tammela[2*]

[1] Drug Research Program, Division of Pharmaceutical Chemistry and Technology, Faculty of Pharmacy, Viikinkaari 5 E (PO Box 56), FI-00014, University of Helsinki, Helsinki, Finland; paula.kiuru@helsinki.fi
[2] Drug Research Program, Division of Pharmaceutical Biosciences, Faculty of Pharmacy, Viikinkaari 5 E (PO Box 56), FI-00014, University of Helsinki, Helsinki, Finland; paivi.tammela@helsinki.fi
[3] Institute of Chemistry, Technische Universität Chemnitz, 09107 Chemnitz, Germany, tobias.rueffer@chemie.tu-chemnitz.de
† Current address of Chinmay Bhat: Government First Grade College, Chamarajanagar (Affiliated to University of Mysore), India
* Correspondence: paivi.tammela@helsinki.fi; Tel.: +358-2941-59628

CONTENTS

1. Syntheses **P2**

2. ^{1}H and ^{13}C NMR spectra of compound 1 and 36 **P5**

3. Single crystal X-ray diffraction measurements **P7**

4. References **P9**

1. Synthesis

General

All reactions were carried out using commercially available starting materials unless otherwise stated. The melting points were measured with Stuart SMP40 automated melting point apparatus and are uncorrected. ^{1}H NMR (300 MHz) and ^{13}C NMR (75 MHz) spectra in $CDCl_3$, d_6-DMSO, or CD_3OD at room temperature were recorded on a Varian Mercury *Plus* 300 spectrometer or Bruker Avance 400 MHz NMR with smart probe. Chemical shifts (δ) are given in parts per million (ppm) relative to the ^{1}H and ^{13}C NMR reference solvent signals ($CDCl_3$: 7.26 and 77.16 ppm; CD_3OD: 3.31 and 49.00 ppm; d_6-DMSO: 2.50 ppm and 39.52, d_6-acetone: 2.05 and 29.84 ppm). Multiplicities are indicated by s (singlet), br s (broad singlet), d (doublet), dd (doublet of doublet), ddd (doublet of doublet of doublets), t (triplet), dt (doublet of triplets), q (quartet) and m (multiplet).The coupling constants *J* are quoted in Hertz (Hz). LC-MS and HRMS-spectra were recorded using Waters Acquity UPLC®-system (with Acquity UPLC® BEH C18 column, 1.7 µm, 50 × 2.1 mm, Waters) with Waters Synapt G2 HDMS with the ESI(+), high resolution mode. The mobile phase consisted of H_2O (A) and acetonitrile (B) both containing 0.1% HCOOH. Microwave synthesis were performed in sealed tubes using Biotage Initiator+ instrument equipped with an external IR sensor. The flash chromatography was performed with Biotage SP1 flash chromatography purification system with 254 nm UV-detector or Biotage Isolera™ Spektra Systems with 200-800 nm UV-detector using SNAP 10, 25, 50 or 100 g cartridges. The TLC-plates were provided by Merck (Silica gel 60-F254) and visualization of the amine compounds was done using ninhydrin staining.

***tert*-Butyl (3-chloropropyl) (methyl) carbamate (15)** [1]

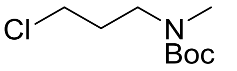

3-Chloro-*N*-methylpropylamine hydrochloride (0.50 g, 3.5 mmol) was dissolved to dry DCM (10 mL) under argon atmosphere. Boc_2O (0.90 g, 4.16 mmol, 1.2 equiv) in DCM (2 mL) and Et_3N (0.96 mL, 7.0 mmol, 2.0 equiv) were added dropwise at 0 °C. The reaction mixture was brought to room temperature in 1 h and stirred further 12 h. It was quenched with a 1 M solution of HCl in H_2O (20 mL) and extracted with DCM (25 mL). The organic layer was further washed with a 1 M solution of HCl in H_2O (2 × 15 mL) and a saturated solution of $NaHCO_3$ in H_2O (3 × 15 mL). The aqueous layers were back-extracted with DCM (20 mL) and the combined organic layer was dried with anh. Na_2SO_4. The crude product was purified by column chromatography (silica gel, isocratic eluent, hexane/EtOAc, 8:2); TLC was stained with iodine. Compound **15** was obtained as a colorless oil (0.76 g, quant.).
^{1}H NMR (300 MHz, $CDCl_3$) δ 3.53 (t, 2H, *J* = 6.6 Hz), 3.34 (t, 2H, *J* = 6.6 Hz), 2.85 (s, 3H), 2.02-1.93 (m, 2H), 1.44 (s, 9H).

***tert*-Butyl [3-[2,6-dibromo-4-[2-[(*tert*-butoxycarbonyl)amino]ethyl]phenoxy]propyl](methyl) carbamate (14)**

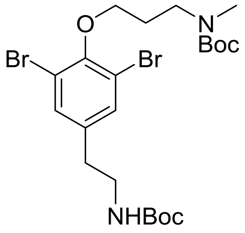

Compound **13** (0.76 g, 1.92 mmol), *t*-butyl (3-chloropropyl) methyl carbamate **15** (0.48 g, 2.31 mmol, 1.2 equiv) and Cs_2CO_3 (0.94 g, 2.88 mmol, 1.5 equiv) were dissolved in DMF (8 mL). The reaction mixture was stirred at 80 °C under argon atmosphere for 24 h, after which it was quenched with a 0.1 M solution of NaOH in H_2O (20 mL). The reaction mixture was extracted with EtOAc (4 × 30 mL), the combined organic layers were dried over Na_2SO_4 and concentrated *in vacuo*. The crude product was purified by column chromatography, Biotage SNAP Cartridge KP-Sil 50 g, gradient elution: (hexane/EtOAc, 32→46%) to give **14** as a white amorphous solid (0.89 g, 83%). ^{1}H NMR (300 MHz, $CDCl_3$) δ 7.32 (s, 2H), 4.01 (t, 2H, *J* = 12.9 Hz), 3.47 (t, 2H, *J* = 14.1 Hz), 3.32 (q, 2H, *J* = 6.9 Hz), 2.92 (s, 3H), 2.71 (t, 2H, *J* = 7.2 Hz), 2.13-2.03 (m, 2H), 1.45 (s, 9H), 1.43 (s, 9H); ^{13}C NMR (75 MHz, $CDCl_3$) δ 155.9, 152.0, 137.8, 133.0, 118.3, 79.5, 71.4, 46.3, 41.6, 35.1, 34.7, 28.6, 28.5.

3-[4-(2-Aminoethyl)-2,6-dibromophenoxy]-*N*-methylpropan-1-amine (6)

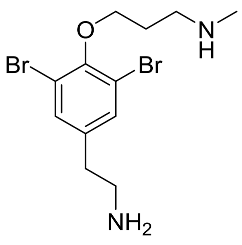

A solution of **14** (2.00 g, 3.62 mmol) in DCM (10 mL) was stirred with TFA (1.5 mL, 1.97 mmol, 5.5 equiv) under argon atmosphere for 48 h at rt. The reaction mixture was then concentrated under reduced pressure to remove excess TFA and DCM. The crude yellow oil obtained was treated with a saturated solution of $NaHCO_3$ in H_2O (2 mL); the addition was monitored by using pH paper; addition of excess $NaHCO_3$ should be avoided as the product is water soluble. The resulting mixture was extracted with EtOAc (2 × 20 mL). The organic layer was dried over anh. Na_2SO_4 and concentrated under reduced pressure to give **6** as a yellow solid (1.52 g, quant.). ^{1}H NMR (300 MHz, CD_3OD) δ 7.47 (s, 1H), 7.43 (s, 1H), 4.06–4.02 (m, 2H), 3.24–3.21 (m, 2H), 2.93–2.88 (m, 2H), 2.71–2.67 (m, 2H), 2.45 (s, 3H), 2.10–2.02 (m, 2H); ^{13}C NMR (75 MHz, $CDCl_3$) δ 153.0, 137.7, 134.4, 119.2, 71.4, 48.3, 41.3, 33.7, 33.1, 27.7.

2-Amino-3-(3,5-dibromo-4-methoxyphenyl)propanoic acid hydrochloride (9) [2]

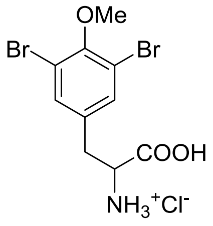

O-Methyl-L-tyrosine **7** (0.50 g, 2.6 mmol) in a 2 M solution of HCl in H_2O (20 mL) was cooled to 0 °C. Bromine (1 mL) was added dropwise, and the reaction mixture was further stirred at rt overnight. It was concentrated to dryness by air, and the resulting solid was washed with *n*-hexane to obtain crude compound **9** as a brown solid (1.29 g, quant.).

Methyl (*S*)-2-amino-3-(3, 5-dibromo-4-methoxyphenyl)propanoate (10) [2]

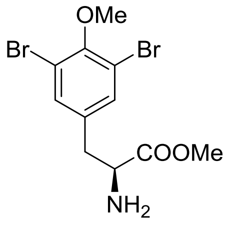

To a stirred solution of amino acid **9** (1.1 g, 2.8 mmol) in MeOH (15 mL) at 0 °C, was added $SOCl_2$ (0.5 mL, 5.60 mmol, 2.0 equiv). The mixture was stirred 30 min at 0 °C and then refluxed for 5 h. The reaction mixture was quenched with a saturated solution of $NaHCO_3$ in H_2O (15 mL) and extracted with EtOAc (3 × 30 mL). The combined organic layer was dried over anh. Na_2SO_4 and concentrated under reduced pressure to give **10** as a pale yellow solid (1.04 g, 93%). ^{1}H NMR (300 MHz, $CDCl_3$) δ 7.36 (s, 2H), 3.87 (s, 3H), 3.73 (s, 3H), 3.69 (br s, 2H), 3.02-2.96 (m, 1H), 2.79-2.77 (m, 1H).

Methyl (*E*)-3-(3,5-dibromo-4-methoxyphenyl)-2-(hydroxyimino)propanoate (11) [2]

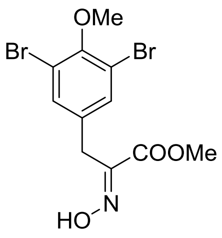

To a well-stirred solution of **10** (0.90 g, 2.54 mmol) in EtOH (8 mL), was added $Na_2WO_4{\cdot}2\,H_2O$ (0.83 g, 2.54 mmol), 30% H_2O_2 (2.5 mL), and water (4.0 mL) at 0 °C. The reaction mixture was further stirred for 14 h at room temperature. It was then quenched with a saturated solution of NH_4Cl in H_2O (10 mL) and extracted with EtOAc (3 × 25 mL). The combined organic layers were dried over Na_2SO_4 and concentrated *in vacuo.* The crude product was purified by column chromatography (silica gel, isocratic elution, DCM/MeOH, 8:2) to give **11** as a white solid (0.38 g, 40%). ^{1}H NMR (300 MHz, CD_3OD) δ 7.47 (s, 2H), 3.38 (s, 2H), 3.82 (s, 3H), 3.80 (s, 3H); ^{13}C NMR (75 MHz, CD_3OD) δ 165.6, 154.0, 150.6, 137.0, 134.3, 118.6, 61.0, 52.9, 30.0.

(*E*)-3-(3, 5-Dibromo-4-methoxyphenyl)-2-(hydroxyimino)propanoic acid (5) [2]

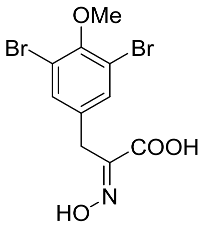

To a solution of **11** (0.14 g, 0.37 mmol) in THF (12 mL) and H_2O (1:1), LiOH (0.13 g, 5.43 mmol, 15.0 equiv) was added and the mixture was stirred for 14 h. The reaction mixture was then quenched with a 2 M solution of HCl in H_2O (until neutral) and was extracted with EtOAc (2 × 20 mL). The combined organic layers were dried over anh. Na_2SO_4 and concentrated *in vacuo*. The pale yellow crude solid was recrystallized from *n*-hexane to give **5** as a white solid (0.12 g, 90%). ^{1}H NMR (300 MHz, d_6-DMSO) δ 12.8 (br s, 1H), 12.4 (br s, 1 H), 7.44 (s, 2H), 3.77 (s, 5H); ^{13}C NMR (75 MHz, CD_3OD) δ 164.8, 151.8, 149.2, 136.1, 132.6, 117.1, 60.3, 28.6.

Unsuccessful coupling attempts towards purpurealidin I 1

Initially, the direct condensation of hydroxylamine ester **11** was tried using amine **6** without success. The general experimental procedure employed was as follows: Carboxylic acid **5** (0.10 g, 0.26 mmol), **6** (0.29 g, 0.78 mmol, 3.0 equiv) and K_2CO_3 (0.11 g, 0.78 mmol, 3.0 equiv.) were charged to a 5-mL Biotage tube pre-flushed with argon. MeOH (3 mL) was then added and the tube was sealed. The reaction mixture was heated in an oil bath at 60 °C. Different work-up procedures were tried after running different batches of reaction (*e.g.* washing with water, dil. HCl, saturated $NaHCO_3$, and 1 M NaOH). In every trial, the product **1** was characterized by ^{1}H NMR, but never obtained as a pure substance.

Table S1. Coupling attemps towards purpurealidin I **1**

**Carboxylic acid 5 (equiv.)**	**Amine 6 (equiv.)**	**Conditions (carried out in a sealed tube)**	**Result**
1.0	3	MeOH, K_2CO_3, 80 °C, 48 h	Mixture
1.0	0.5	MeOH, K_2CO_3, 80 °C, 48 h	Mixture
1.0	3.0	DMF, 80 °C, 48 h	Mixture
1.0	3.0	MeOH, K_2CO_3, 65 °C, 2 h, MW	Mixture
1.0	3.0	MeOH, K_2CO_3, 80 °C, 48 h	48% crude yield. Product **1** seen in ^{1}H NMR, the purification failed
1.0	0.5	MeOH, *t*-BuOK, 80 °C, 48 h	Mixture

2. NMR spectra

2.1 ^{1}H and ^{13}C NMR spectra of purpurealidin I **1**

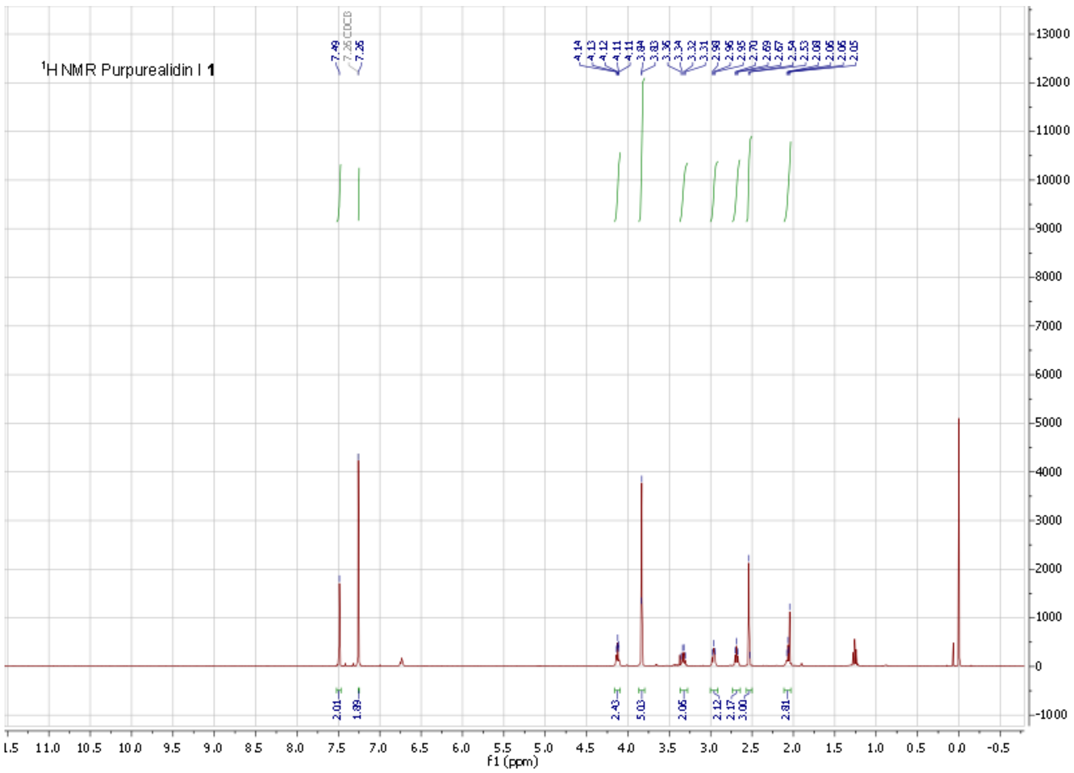

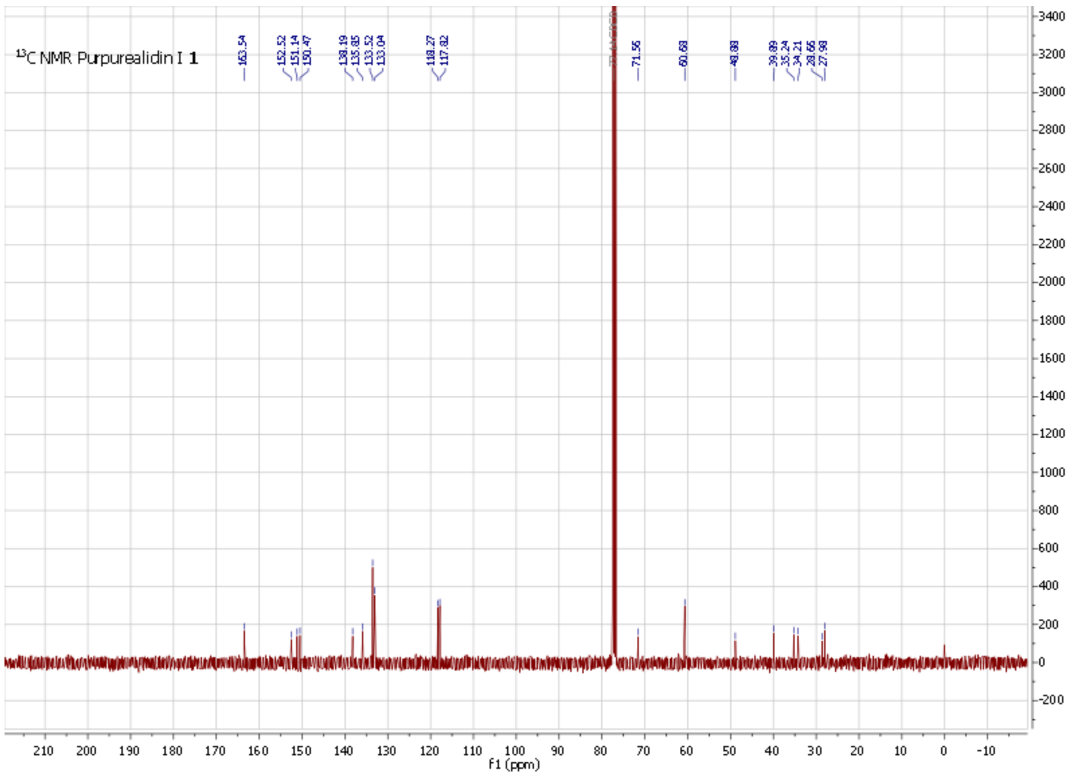

2.2 ^{1}H and ^{13}C NMR spectra of compound **36**

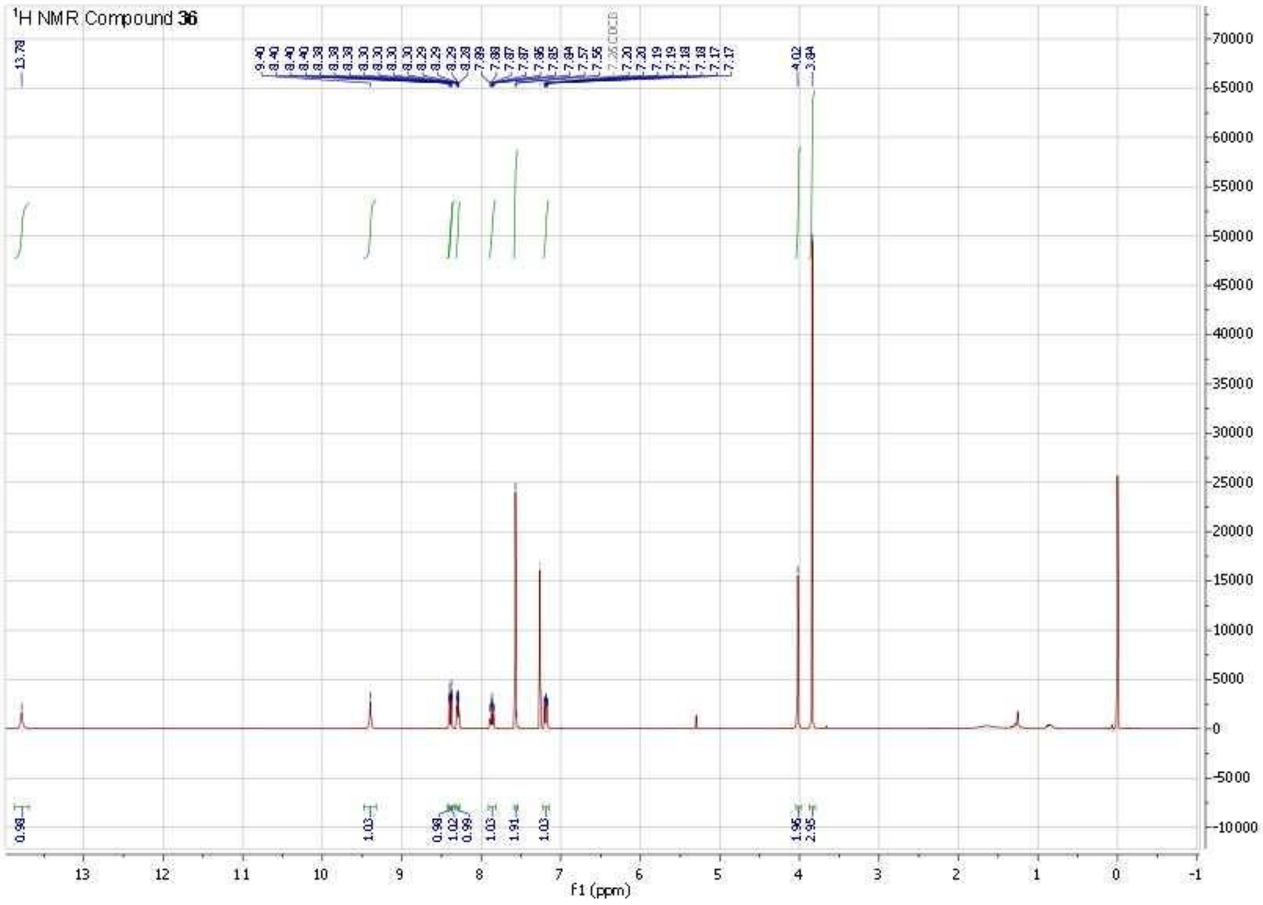

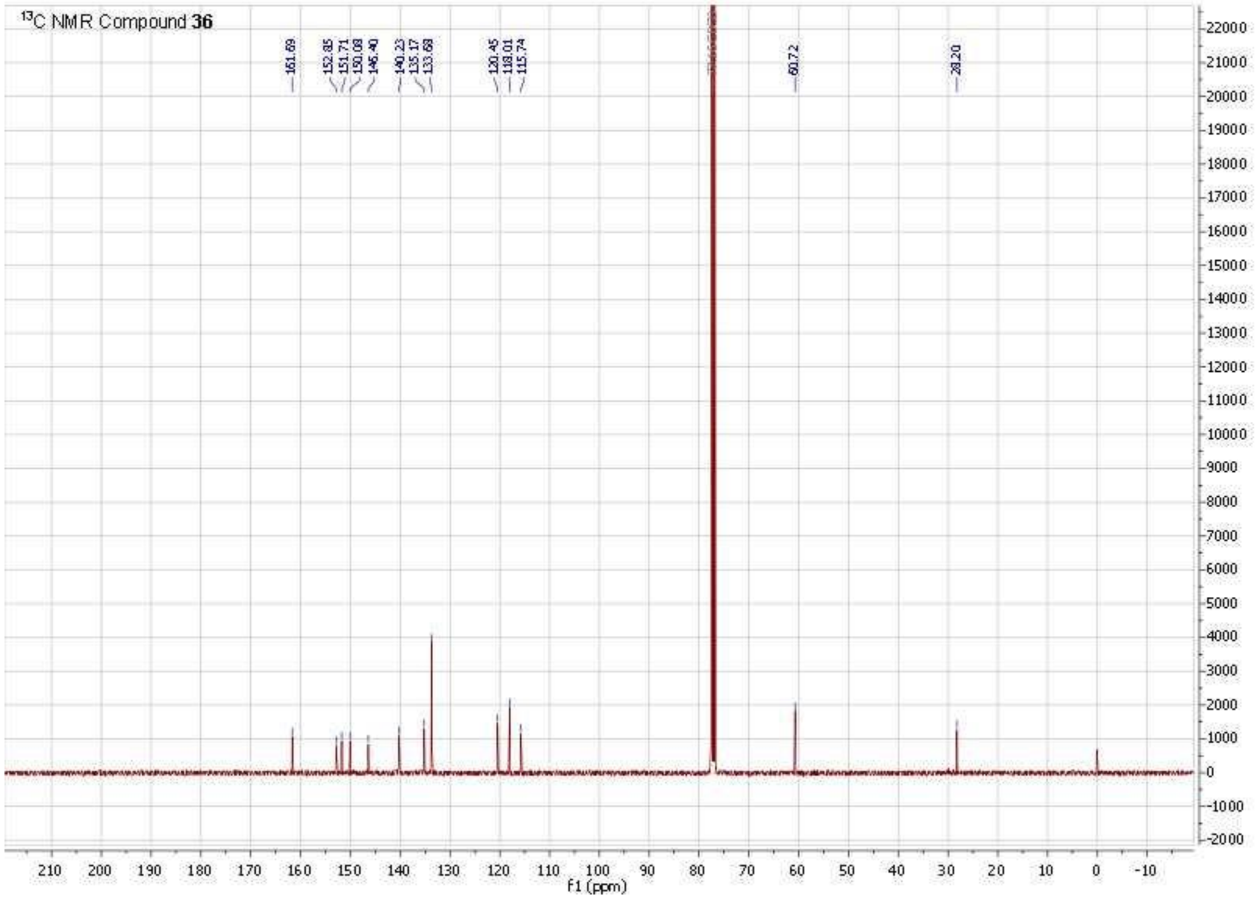

3. Single crystal x-ray analysis

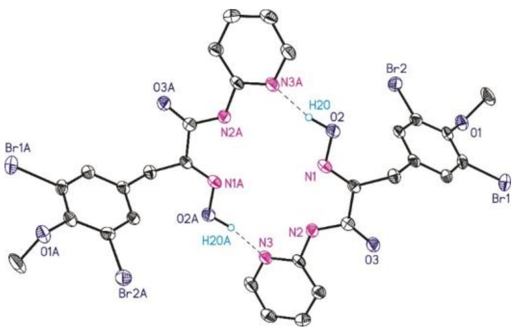

Figure S1. ORTEP (50% probability ellipsoids) of the dimer formed by **36** in the solid state due to formation of intermolecular hydrogen bonds, indicated with dotted lines. All carbon- and nitrogen bonded hydrogen atoms and packing solvent molecules have been omitted for clarity. Symmetry code: “A” = –1 – *x*, 1 – *y*, 1 – *z*.

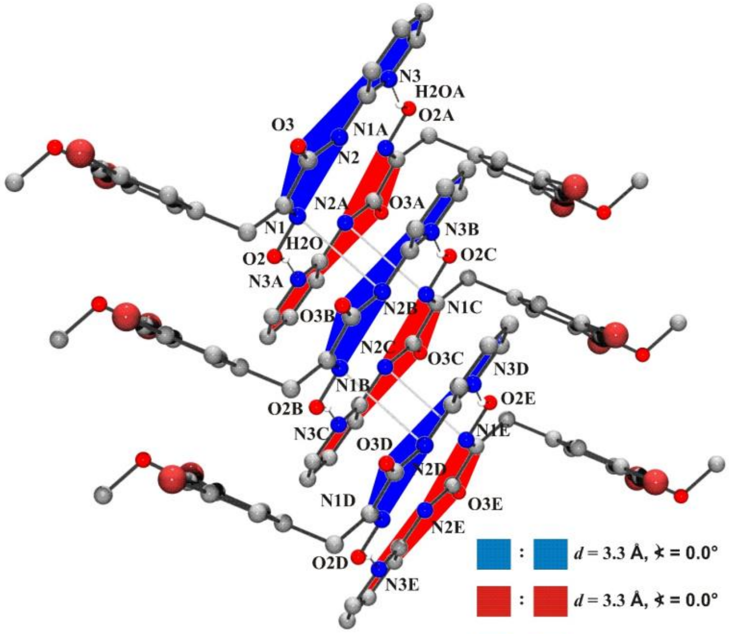

Figure S2. Ball-and-stick model of a representative part of one of the bands formed by **36** in the solid state due to formation of intermolecular hydrogen bonds indicated by dotted lines and dispersion interactions. The sign *d* refers to the averaged distance of mean planes of atoms belonging to blue or red shaded areas, while the sign ∢ refer to the interplanar angles. Grey lines indicate the shortest intermolecular distances of 3.272 Å due to dispersion interactions between nitrogen atoms N1 and N2B as well as symmetry related atoms. Symmetry codes: “A” = –1 – *x*, 1 – *y*, 1 – *z*. “B” = –*x* + 1, *y*, *z*. “C” = –*x*, 1 – *y*, 1 – *z*. “D” = –*x* + 2, *y*, *z*. “E” = –*x* + 1, 1 – *y*, 1 – *z*.

D–H···A	D–H	H···A	D···A	D–H···A
Intra N2–H2···N1	0.86	2.22	2.637(4)	109
Inter O2–H2O···N3	0.97(3)	1.74(3)	2.687(4)	164(4)

CCDC 1873696 contains the detailed crystallographic data of this report. This data are available free of charge via the Cambridge Crystallographic Data Centre:
www.ccdc.cam.ac.uk/data_request/cif .

Summary of Data CCDC 1873696

Compound Name:
Formula: C15 H13 Br2 N3 O3,C1 H1 Cl3
Unit Cell Parameters: a 4.4118(3) b 24.7517(14) c 18.6721(11) P21/n
----

4. References

[1] Buchholz, M.; Heiser, U.; Schilling, S.; Niestroj, A.J.; Zunkel, K. Demuth, H.-U. The First Potent Inhibitors for Human Glutaminyl Cyclase: Synthesis and Structure-Activity Relationship. *J. Med. Chem.* **2006**, *49*, 664-677. DOI**:** 10.1021/jm050756e

[2] Hillgren, J.M.; Öberg, C.T.; Elofsson, M. Syntheses of pseudoceramines A–D and a new synthesis of spermatinamine, bromotyrosine natural products from marine sponges. *Org. Biomol. Chem*. **2012**, *10*, 1246-1254. DOI: 10.1039/C1OB06722B

[3] García, J.; Pereira, R.; de Lera, A.R. Total synthesis of the natural isoprenylcysteine carboxyl methyltransferase inhibitor spermatinamine. *Tetrahedron Lett.* **2009**, *50*, 5028-5030. https://doi.org/10.1016/j.tetlet.2009.06.087

[4] Kottakota, S.K.; Evangelopoulos, D.; Alnimr, A; Bhakta, S; McHugh, T.D.; Gray, M.; Groundwater, P.W.; Marrs E.L.; Perry J.D.; Spilling C.D., Harburn J.J. Synthesis and biological evaluation of purpurealidin E-derived marine sponge metabolites: Aplysamine-2, aplyzanzine A, and suberedamines A and B. *J Nat Prod.* 2012, 75, 1090-1101. DOI: 10.1021/np300102z
